# Genetics of conditional extended mycelial cell viability of *Streptomyces minutiscleroticus* in deep starvation phase implicates the involvement of (p)ppGpp, *clpX*, and a histidine kinase *sasA*

**DOI:** 10.3389/fmicb.2024.1495007

**Published:** 2024-11-14

**Authors:** Vaidehi Chatupale, Jayashree Pohnerkar

**Affiliations:** Department of Biochemistry, The Maharaja Sayajirao University of Baroda, Vadodara, India

**Keywords:** LTSP, longevity, stationary phase, *clpX* protease, *sasA* histidine kinase

## Abstract

Bacterial lifespan ranges from a few hours to geological timescales. The prolonged survival trait under extreme energy starvation is essential for the perpetuation of their existence. The theme for long-term survival [long-term stationary phase (LTSP)] in the non-growing state may be dependent on the diversity in the environmental niche and the lifestyle of the bacteria, exemplified by longevity studies, albeit few, with model organisms. In the present study, we characterized the LTSP of mycelial cells of *Streptomyces Minutiscleroticus*, which remain metabolically active, demonstrate ongoing protein synthesis—killed by protein synthesis inhibitors—and remarkably by the cell-wall synthesis inhibitors, vancomycin, and ampicillin, suggesting “growth.” Their rapid turnover is also evident in ~10-fold loss of colony-forming unit (CFU) over a year, suggesting that for the death of one “old” cell, slightly less than one “new” cell is born. This longevity is consequent to (i) induction of the gene expression program effected by non-metabolizable, non-ionic osmolyte, sucrose, thus conditional, and (ii) possibly rendering this carbon utilizable by the production of a slow hydrolytic activity generating glucose, reinforcing the relevance of low-level energy resource for long term survival in the starvation phase. The viability parameters of LTSP cells measured through up to 90 days suggest that the stationary phase transitioning into LTSP following nutrient exhaustion is nearly quantitative. Expectedly, the viability in LTSP is (p)ppGpp/RelA dependent. Whereas mutation in chaperone *clpX*, negatively affects survival in stationary phase, overexpression of signal sensor-transducer histidine kinase, SasA8, enhances cell survivability. The relevance of longevity functions identified here requires further deduction of the genetic program.

## Introduction

Bacteria inhabit virtually the entire biosphere, leaving scarcely any environment considered uninhabitable. They are subject to extremes of environmental stresses, both biotic and abiotic, such as starvation for nutrients, variation in temperature, pH, and salinity. Their resilience in adjusting to these extremes is remarkable and necessitates scientific appreciation (Hazan et al., 2021; Hoehler and Jørgensen, 2013; Bergkessel et al., 2016; Dworkin and Harwood, 2022). Although, it may not be possible to explore the long-term stationary phase (LTSP) of bacteria surviving in nature in their ecological settings, simulating their lifestyle in the lab setting is an alternative. From among the different states of growth phases of bacteria, the extended stationary phase appears relevant to understanding the physiology and metabolism of the most dominant form of their existence (Hoehler and Jørgensen, 2013; Bergkessel et al., 2016; Dworkin and Harwood, 2022). Although this strategy requires studying bacteria in isolation, in the batch culture, and in pure homogeneous form rather than in their community existence which can be unrealistic, the investigations with model bacteria have been undertaken in the hope that the knowledge of their survival in extreme energy-starved state could account for their survival in nature, albeit with some limitations. It is proposed that the energy calculation of the metabolically starved bacteria in nature is vastly lower than a typical culture of bacteria studied even for the estimate of maintenance function under the defined conditions; moreover, given the number of cells in the batch culture entering LTSP in the lab experiments, it is not clear the extent to which the death occurring in batch cultures is part of microbial life under low energy (Hoehler and Jørgensen, 2013). Nonetheless, research with diverse bacterial non-growing states living in different niches and with varying lifestyles will provide a glimpse of variations in the available information using the model systems (Bergkessel et al., 2016).

Adaptation of bacteria to energy limitation and persistence under that condition is beginning to be probed in some detail over the past two decades in the lab (Hazan et al., 2021; Hoehler and Jørgensen, 2013; Bergkessel et al., 2016; Dworkin and Harwood, 2022). Even though specific bacterial genera, such as *Bacillus*, *Streptomyces*, and *Clostridia*, form spores, the differentiated cell type that can withstand adverse environmental stresses, it is equally common that non-sporulating bacteria, too, live long in the absence of nutrients.

Discussed as under is a brief overview of the genetic basis of survival strategies in LTSP of some non-sporulating model bacteria to provide perspective of the field. Longevity studies with *Bacillus subtilis* non-sporulating mutant attempted to examine genetic factors responsible for the prolonged viability of a small fraction of surviving cells in a non-growing state among a large population of dead cells. The characteristics of these cells include increased metabolism, reduced size, and sustained protein synthesis (Gray et al., 2019). The “growth” of the morphologically altered cell has been demonstrated in this study. Though the transcriptome of the cell is found to be different from that of the stationary/exponential cells, genetic mutations in several candidate genes have no appreciable effect on LTSP, except for the multiple protease deficient strain, implicating protein turnover to be essential for long-term survival (Gray et al., 2019). In the prototype model *Escherichia coli*, studies revealed genetic mutations in a small number of survivors that overtake a population in the growth-arrested state at the expense of the death of the non-mutant majority. At every cycle of growth and death of evolving culture, mutations in genes affecting several functions such as DNA repair, replication, metabolic functions, transporters, and global regulatory proteins (Finkel, 2006; Kram et al., 2020; Zambrano et al., 1993) have been found to confer a growth advantage in the stationary phase (GASP). The viability of carbon-starved and stationary phase cells of *Pseudomonas aeruginosa* is dramatically affected by *ftsH* acting as a protease rather than by its primary effect of increasing LPS synthesis to toxic levels. The impact on growth by other proteases and stresses such as heat and pH exacerbate the growth defects of *ftsH* mutant, indicating a defect in the removal of accumulated proteins under stress conditions is the reason for lethality (Basta et al., 2020). Similar studies with some pathogenic bacteria, such as *Brucella suis*, *Acinetobacter*, and *Mycobacterium tuberculosis* show that the differences in metabolic and regulatory genes are evident in the proteasome analysis of LTSP survivors (Dahouk et al., 2013; Lostroh and Voyles, 2010; Betts et al., 2002). The long-term viability of carbon-limited/nutrient-deprived cells of *Rhodopseudomonas palustris* has been shown to be dependent on adenosine triphosphate (ATP) and expression of (p)ppGpp-dependent stationary phase transcriptome and gene functions involved in viability which include *era* and *RNase* (Pechter et al., 2017; Yin et al., 2019). In contrast to the unique property of *R. palsutris* to generate ATP (by photophosphorylation) independent of nutrient availability, in other examples of bacteria, ATP generation is dependent on nutrient utilization. Appreciation of the variation in the strategies is possible with studies involving diverse bacteria inhabiting different environments and life regimes.

In the study reported here, we describe long-term surviving cells of the soil organism *Streptomyces minutiscleroticus*, a Gram-positive, spore-forming, obligate aerobe, non-pathogenic, and producer of the antibiotic chromomycin (Prajapati et al., 2019). A model for studies on development and differentiation in bacteria, the distinct phases of *Streptomyces* growth on solid agar/submerged state consists of the initial growth of spores into a compartmentalized mycelium (MI) that matures into mycelial differentiation stage II (MII) following a short nongrowing phase of cell death (PCD). The multinucleated hyphae in the MII phase undergo a second round of massive programmed cell death to give rise to aerial hyphae on solid agar surfaces that further differentiate them into spores. In the submerged cultures, however, the events post-MII phase leading to sporulation are apparently lacking, except for some strains of *Streptomyces* (Dissel et al., 2014; Manteca et al., 2008). Although *Streptomyces coelicolor* cells retain viability for an extended period when subjected to anaerobic stress under nutrient-replete conditions (Keulen et al., 2007), our study, to our knowledge, is the only study on LTSP of hyphal growth addressing the major limitation of a reliable quantification method for measuring viability, unlike studies with other model bacteria, where the cell growth is incidentally, planktonic. However, similar studies with different species of *Streptomyces* are required to understand the widespread prevalence of this phenomenon. Additionally, bacterial population in the deep stationary phase features substantial phenotypic heterogeneity possible to be studied with the recent microfluidic and FACS analyses techniques designed for single-cell study that is challenging with the hyphal cells of *Streptomyces* (Bergkessel et al., 2016).

Notwithstanding the inadequacies, we investigated the conditional LTSP state of *S. minutiscleroticus* mycelial cells in the submerged growth in batch culture using multiple approaches, including genetic. We identified and validated, by gene disruption and multicopy expression studies, the importance of (p)ppGpp synthetase *relA*, *clpX* encoding chaperone, and histidine kinase, *sasA8*, affecting the long-term viability of mycelial cells.

## Materials and methods

### Growth of Streptomyces

*Streptomyces* cells were cultured in Tyrptone soya broth (TSB) medium containing 17 g of pancreatic digest of casein, 3-g peptic digest of soybean, 5-g sodium chloride, 2.5-g glucose per liter. Yeast extract malt extract (YEME) medium consists of (yeast extract 3 g, malt extract 3 g, peptone 5 g, glucose 10 g, and sucrose 34% final concentration per liter). The high osmolarity TSB + YEME medium used in this study contains a 1:1 proportion of TSB and YEME. Other media used in this study are R2YE (*Streptomyces* growth medium), R4 (R2YE minus yeast extract and CAA), SMA (2% soybean, 2% mannitol, 2% agar), and supplemented minimal medium agar (SMMA) are described in reference (Kieser et al., 2000).

### Measurement of sucrose hydrolytic activity by glucose estimation in the cell extract of TSB + 20% sucrose grown cells

Approximately 1-ml cells were harvested and washed twice with 0.1-mM ammonium acetate buffer pH 6. 250-μl sonicated cell extract was mixed with 250 μL of 1% sucrose solution as a substrate and kept for 22 h at 37°C. The glucose oxidase-peroxidase coupled method (GOD-POD) (glucose 5 min, RECKON DIAGNOSTICS P. LTD) method was used to estimate the glucose amount generated from this reaction according to the kit’s instructions. The absorbance was measured at 505 nm.

### Resazurin assay

Approximately 1 mL of cells washed in 0.9% (w/v) N-saline was added with resazurin dye added at the final concentration of 30 μg/mL and incubated in the dark for 30 min. The conversion of blue resazurin to pink resorufin was measured in a fluorimeter at every 5 min interval for 30 min at the excitation at 530 nm and the emission at 590 nm. The fluorescence intensity was divided by protein (mg/ml) for normalization. Normalized fluorescence of resazurin of exponential cells (grown for 2 days in TSB) as control was set at 1, and the normalized intensity of test cells (cultured for different days) was plotted relative to the control.

### Measurement of PMF (membrane potential) by DiOC6(3)

Accumulation of the membrane potential fluorophore, 3,3′-dihexyloxacarbocyanine iodide, DiOC6(3) (Sigma–Aldrich Catalog No. 318426) by viable cells, including bacterial cells, reflected as green fluorescence is directly proportional to the magnitude of proton motive force (PMF). Approximately 1 mL of cells were washed twice with 0.9% (w/v) N-saline and resuspended in 0.5 mL of the same. DiOC6(3) dye was added at 50 nM final concentration (5 μL of 10 μM stock solution), and fluorescence was recorded at excitation 482 nm and emission at 504 nm. The fluorescence reading was divided by protein concentration (mg/ml) for normalization.

### Proton motive force is essential for viability

The proton ionophore uncoupler carbonyl cyanide chlorophenylhydraz (CCCP) dissipates membrane potential and inhibits growth, evident as a decrease in CFU following the treatment. For measuring loss of viability with CCCP treatment, 1 mL of cells were washed with 0.9% (w/v) N-saline. CCCP (dissolved in DMSO) was added at the final concentration of 30 μM. After 48 h of incubation, an equal amount of CCCP-treated cells were both used for plating for CFU count and for measuring DiOC6(3) fluorescence (as above). The CCCP untreated cells were included in the plating for comparison.

### Monitoring PMF by sensitivity to aminoglycoside antibiotic streptomycin

Uptake of aminoglycoside antibiotics has been shown to be PMF dependent (Allison et al., 2011; Taber et al., 1987; Davis, 1987; Bruni and Kralj, 2020). One milliliter cells were either washed and resuspended in 0.9% (w/v) saline or directly treated with streptomycin at the final concentration of 50 μg/mL for 48 h. Viability was quantified by plating appropriate dilutions on R2YE agar. The untreated control cells were plated for comparison.

### Measurement of sensitivity of LTSP cells to vancomycin/ampicillin

Approximately 1-ml cells were either washed and resuspended in saline or directly treated with vancomycin/ampicillin at a final concentration of 50 μg/mL for 48 h. Appropriate dilutions were plated on R2YE agar for monitoring viability and compared with the untreated control cells.

### Measurement of caseinolytic activity in *clp* mutants

One milliliter of culture harvested from the exponential phase of WT (JP2), *clp*X mutant (JP4), JP4/pVM2 (*clp*X complemented strain), and *clp*P1 mutants were washed twice in 0.9% (w/v) N-saline. 1% of casein was added to the cells, and turbidity was measured spectrophotometrically at 600 nm. This was considered as 0 time point. Caseinolytic activity of *clp* mutants was calculated by a decrease in turbidity of casein solution at every 4 h interval.


$$\begin{array}{l} \mathrm{Units of activity}={OD}_{600}\;t\left(0\right)-{OD}_{600}\;t\\ \left(4\;h,8\;h,12\;h\right)/t\left(4\;h,8\;h,12\;h\right)\times \mathrm{Volume of the culture}\text{.} \end{array}$$

Specific activity is denoted as units/mg protein.

### Growth curve

A growth curve experiment was performed for cells cultured in low osmolarity TSB broth and high osmolarity TSB + 20% sucrose medium. The initial spore inoculum size was adjusted to ~10^6^ CFU/mL. One milliliter culture was harvested once every 2 h, washed twice with 0.9% (w/v) N-Saline, resuspended in the same, and boiled at 95°C for 10 min, and protein estimation was performed by the Folin Lowry method. The CFU count of each time point was performed by plating at appropriate dilution. A low osmolarity culture sample was collected for up to 1 week, and the growth curve of a high osmolarity culture was carried out for up to 60 days.

### Green fluorescent protein measurement assay

pIJ8655 contains the eGFP gene under the control of thiostrepton inducible promoter (Sun et al., 1999). The plasmid DNA was integrated into the WT *Streptomyces* genome at the *att* site by intergenic conjugation. GFP fluorescence was quantitated by inducing GFP expression with thiostrepton at 50 μg/mL final concentration for 48 h, once every 15 days. One milliliter of the thiostrepton-treated cells were washed by 0.9% (w/v) N-saline twice, and resuspended in the same. GFP fluorescence reading was recorded at excitation 488 nm and emission at 500 nm.

### Viability measurement

CFU count—cells were plated on R2YE agar by spreading appropriate dilutions of cells and counting CFUs after 48 h of incubation at 30°C.

By staining cells with “LIVE/DEAD Bac-Light Bacterial Viability Kit”—0.1-ml cells were washed with 0.9% (w/v) NaCl, and 20 μL of suspension was treated with “LIVE/DEAD Bac-Light Bacterial Viability Kit” (Molecular Probes), for 10 min in the dark according to manufacturer instructions. A mixture of Syto9 and propidium iodide (PI) dyes differentially stain live cells, green and dead cells, red. Suspension of cells was taken on the slide to observe under Leica TCSSP2-AOBS confocal laser-scanning microscope and LSM—710 confocal microscopes at wavelengths of 488 nm (green) and 568 nm (red) excitation, and 530 nm (green) or 630 nm (red) emission.

### Viability by fluorimeter

Approximately 0.5-ml cells were washed with 0.9% (w/v) NaCl, and 100-μl cells were stained with 100 μL of Syto9/PI mixture (“LIVE/DEAD Bac-Light Bacterial Viability Kit L13152) for 20 min. The final concentration of each dye was 6-μm Syto9 and 30-μm PI, which is prescribed for quantitative estimation of live/dead cells by fluorimeter. Fluorescence readings were recorded for PI at excitation 480 nm and emission 500 nm, and for Syto9 at excitation 545 nm and emission 610 nm. The ratio of Syto9/PI was used as a measure of viability.

### Measurement of ATP levels

Approximately 1-ml cells were harvested and washed with 0.9% (w/v) NaCl twice and resuspended in the same. Sonication was carried out for 1 min at 15-s pulse on and 5-s pulse off, at 20% amplitude. Approximately 100-μl of cell extract was used to estimate ATP by ATP Determination Kit (A22066, Thermofisher Scientific), according to manufacturer instructions. The readings were taken on a multimode reader. The standard graph was plotted using different ATP concentrations (1–5 nM). The readings were normalized by protein concentration mg/ml.

### Reverse transcription polymerase chain reaction

Bacterial cells were collected by centrifugation on different days of the growth, that is, day 2, day 5, and day 90 at 4°C. After a brief lysozyme treatment (0.5 mg/mL for 5 min at 37°C), total RNA was extracted by using phenol/guanidine thiocyanate mixture (Trizol reagent, Invitrogen), according to the manufacturer’s protocol. Quality and quantity were confirmed by visualizing RNA on agarose gel and acquiring an absorbance ratio of 260/280, respectively. Residual DNA was removed by treating with DNAse (Ambion). PCR was performed using *pfk2* primers to assess the removal of DNA contamination. An equal amount of RNA, that is, 2 μg was used from all samples for RT-PCR (Bio-Rad kit). The cDNA thus prepared was directly used for expression studies (30 PCR cycles) using *pfk2* as an internal control.

*pfk2* was chosen as an internal control because the amount of transcript was the same between the cells grown to different days in either a low osmolarity or high osmolarity medium. Reads per kilobase per million mapped reads (RPKM) of *pfk2* is almost exactidentical between 5 days cells and 90 days cells (in 5 days, RPKM was 1756; in 90 days, RPKM was 2,769; and the *p*-value was 0.1277).

### Transcriptome analysis by RNA sequencing

RNA extraction for RT-PCR and RNA sequencing—cells of *S. minutiscleroticus* were grown in low osmolarity TSB broth for 5 days and in high osmolarity TSB + 20% sucrose for 90 days at 30°C under constant shaking. Since the 90-day culture was highly viscous and contained dispersed growth, cells from the 10-ml culture were washed thoroughly to remove sucrose. This pellet size matched with 2-ml cells of 5-day-old culture. RNA was extracted from cells pre-treated with lysozyme (1 mg/mL) for 10–15 min using phenol/guanidine thiocyanate mixture and Trizol reagent (Invitrogen), according to manufacturer’s protocol. RNA was quantitated by 260 nm/280 nm ratio using the ultraviolet (UV) spectrophotometer also for assessment of its purity.

A part of this RNA sample was sent to Clevergene Biocorp Private Limited Bengaluru, Karnataka, India, for RNA sequencing and transcriptome analysis. Ribosomal RNA (rRNA) was depleted using QIAseq FastSelect-5S/16S/23S Kit (Catalog No. 335925, Invitrogen) according to the manufacturer’s protocol. *de novo* transcriptome was assembled using Rockhopper2 with default parameters. The assembled contigs were clustered using cd-hit-est with default parameters. The QC passed reads were mapped to the assembled transcriptome using Bowtie2. For differential expression analysis, the DESeq2 package was used. The read counts were normalized, and differential expression analysis was performed. Genes with absolute log2-fold change ≥2 and *p*-value ≤ 0.05 were considered significant. Between 90 days of high-osmolarity culture and 5-days of low osmolarity culture, differentially upregulated genes were 733 and downregulated genes were 554.

### Constructions of plasmids used in this study for the construction of knock-out

The plasmid derivative of pSET152 (Kieser et al., 2000) was used for suicide vector preparation for carrying out gene disruption experiments. 1.8 kb *Hind*III fragment comprising of ΦC31 integrase function involved in site-specific integration of the DNA in the genome was removed by *Hind*III digestion and intramolecular religation to yield pVM1. Approximately 0.4–0.5 kb of internal fragment of the early part of the gene was PCR amplified using relevant primers engineered with sites for *Bam*H1 and *EcoR*1 in their primers (Supplementary Table S2). The PCR DNA was treated with the corresponding restriction enzyme (RE) and ligated to appropriately treat pVM1 DNA. The clones containing the PCR insert were confirmed by RE digestion (using unique RE sites from the insert) and by PCR. Two independent verified clones from the above experiment were transformed into ET12567/pUZ8002, and intergenic conjugation with *S. minutiscleroticus* was carried out using two independent transformants of *E. coli* according to the protocol described in Kieser et al. (2000). Apramycin antibiotic at 50 μg/mL final concentration was used for the selection of exconjugants on SMA plates. Integration of the plasmid into the corresponding open reading frame (ORF) via single crossover homologous recombination disrupts its function, creating the KO mutant. We used over 4–5 independent KO mutants in the growth experiment. The culture’s viability was verified independently at least three times over a period exceeding 1 month.

### Construction of *clpX* expression vector

Full length 1,473 bp PCR of *clp*X consisting of 1,406 nucleotide ORF and 67 extra nucleotides from 5′ end of the gene were PCR amplified using *clp*X left and *clp*X right (Supplementary Table S2) digested by *Nde*1 and *Hind* III REs and cloned into pIJ10257 (Supplementary Table S1) at the corresponding sites to yield pVM2. Clone confirmation was carried out by both PCR and RE digestion. This DNA was introduced into the *Streptomyces* strain by conjugation with *ET*12567/pUZ8002/pVM2 *clpX*+. Selection of exconjugants was carried out by resistance to hygromycin (100 μg/mL final concentration).

### Construction of *sasA8* expression vector

Full length 1,523 nucleotide PCR of *sasA8* was PCR amplified using *sasA8* left and *sas*A8 right (Supplementary Table S2), digested by *Nde*I and *Pac*I and cloned into pIJ12551 (Supplementary Table S1) at the corresponding sites. The representative clone was called pVM3. Clone confirmation was carried out by PCR and by RE digestion. This DNA was introduced first in to *ET*12567/pUZ8002 by transformation. Conjugation was performed using *ET*12567/pUZ8002/pVM3 *sasA8*^+^. Selection of exconjugants was carried out by resistance to apramycin (100 μg/mL final concentration).

### Conjugation between *Escherichia coli* and *Streptomyces*

The plasmids intended for conjugation into *Streptomyces* were initially transformed into *E. coli* ET12567/pUZ8002. *E. coli* transformants were grown overnight at 37°C. in Luria broth provided with relevant antibiotics, subcultured 1:100, and grown further for 3–4 h at 37°C. The cells were pelleted, washed thrice with Luria broth (LB), and resuspended in 2-ml LB (donor culture). Spores of recipient *Streptomyces* were collected in 2xYT medium and were induced to germinate by heat shock at 50°C for 15 min. Equal volumes of the donor culture (*E. coli* cells 0.1 mL, 2 × 10^8^ cells/ml) recipient (10^7^ *Streptomyces* spores) were mixed, and 100 μL was plated onto soybean Mannitol agar plates supplemented with 10 mM MgCl_2_. Plates were incubated at 30°C for 16 h and then covered with 1 mL of sterile distilled water containing the appropriate antibiotic. Incubation at 30°C was continued for about a week to allow the outgrowth of the exconjugants.

## Results

### Characterization of long-term stationary phase cells of *Streptomyces minutiscleroticus*: the importance of the subjective CFU metric used for measuring viability in the present studies

At the outset of our study, we acknowledge the inherent challenges of approximation and arbitrariness of the quantitative parameter of growth, the CFU, that had been used to study viability in the stationary phase of the organism *S. minutiscleroticus*. As discussed earlier, the bacterium grows as vegetative branching hyphae containing multicellular multi-nucleoid compartments. This causes the estimates of a number of viable cells (CFU) in the culture to be only approximate and not a correct reflection of the number of cell divisions. The commonly used metrics of both protein content and dry mass used for measuring growth, however, cannot quantify the ability to form a colony, a measure of “life.” Thus, even though the pellet fragmentation during the stationary phase affects CFU counts (Zacchetti et al., 2018), the significance of CFU as a proxy for survival in the stationary phase remains indispensable.

We additionally rejudged the CFU count and viability of the cells using a LIVE/DEAD Bac-Light Bacterial Viability Kit containing Syto9 and PI (Thermofischer L13152). This viability kit quantifies live cells with intact membrane stain green because of the uptake and retention of Syto9 dye and dead cells with damaged membrane stain red as they are permeable to PI (Yagüe et al., 2010). Since the excitation/emission spectra (Syto9 ex. 545 em. 610 nm, PI ex. 480 nm em. 500 nm) of these two dyes are nonoverlapping, the ratio of Syto9/PI fluorescence was used wherever possible to reassess the measurement of viability by CFU.

### An increase in the osmotic strength of the growth medium extended the viability of the mycelial growth of *Streptomyces minutiscleroticus*

**Figure 1:**
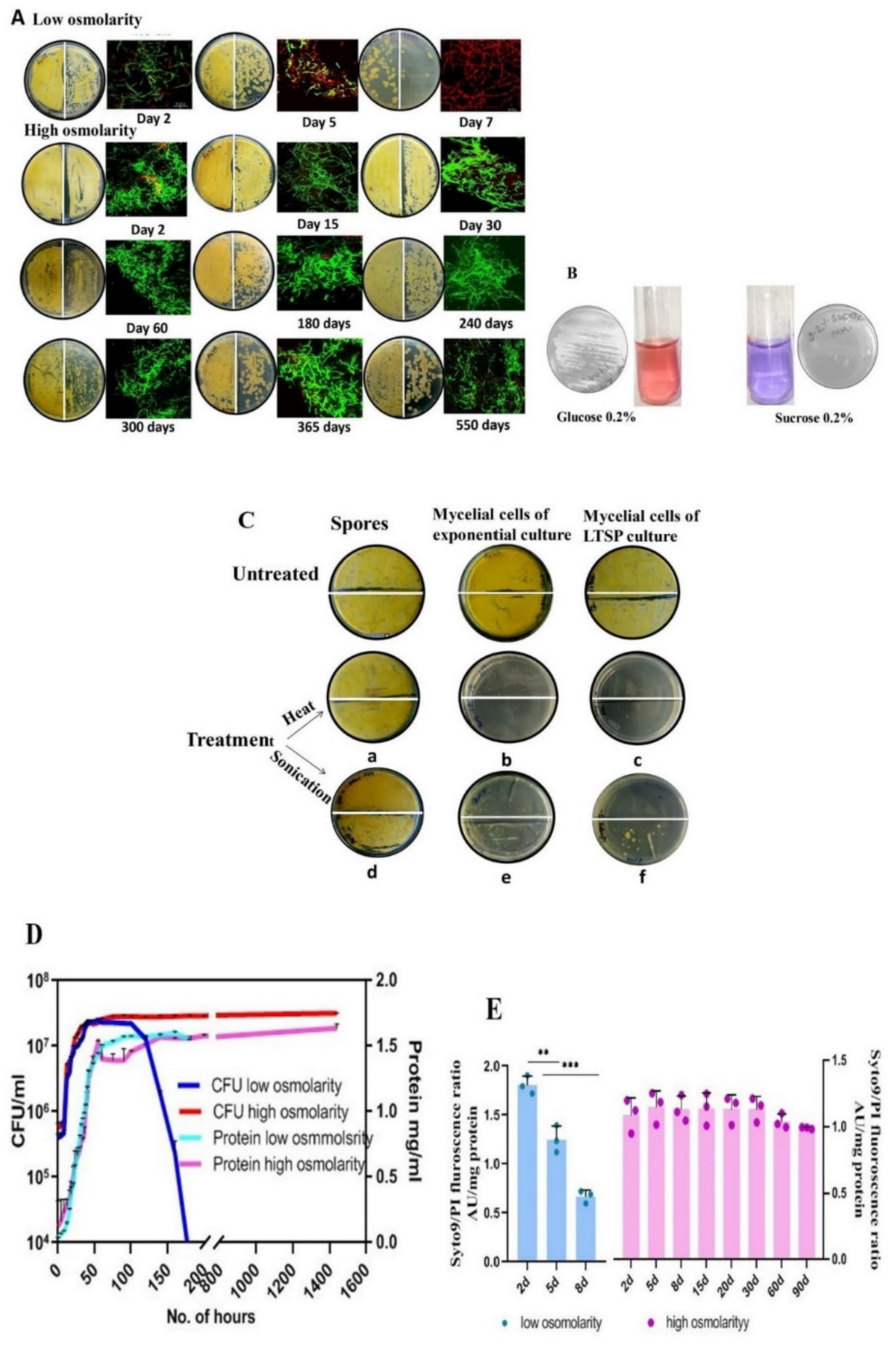
Characterization of LTSP cells of *S. minutiscleroticus*. **(A)** Viability of LTSP cells: Representative images of CFU of *S. minutiscleroticus* cells grown in low osmolar TSB for 7 days and in high osmolar TSB + 20% sucrose medium for ~500 days. 50 μL of cells were plated at 10^0^ and at 10^−1^ dilution on the left-and on the right half of the agar plate, respectively; included against each picture of the agar plate is the confocal merged image of the Syto9/PI-stained cells using LIVE/DEAD Bac-Light Bacterial Viability Kit from Themofisher (L13152). **(B)** The inability of *S. minutiscleroticus* cells to utilize sucrose as the sole carbon source: cells fermenting glucose turn pink in resazurin-supplemented broth; blue color indicates no growth with sucrose as the only carbon source. **(C)** Long-term viable cells are mycelial cells and not spores: Representative images of sensitivity of long-term stationary phase (LTSP) cells (grown in high osmolar medium TSB + sucrose) to heat and to sonication (c, f) similar to the cells of exponentially growing cells (b, e). On the contrary, spores (a, d) are recalcitrant to these treatments. **(D)** Growth curve of *S. minutiscleroticus* cells in media of low and high osmolarity: Growth curve of cells cultivated in low osmolar TSB and high osmolar TSB + 20% sucrose broth. Representative data of at least two independent growth experiments is plotted using CFU count (left y-axis) and cellular protein content (right y-axis). Note the CFU count of the cells grown in low osmolar TSB shows a strong decline on 7th day; the protein content may continue to remain constant for an extended time (not shown). On the contrary, the CFU and protein content plots of high osmolarity cultures run parallel for up to 90 days of the experiment. **(E)** Assessing viability by fluorescence ratio of Syto9/PI-stained cells cultivated in low and high osmolar growth media: Fluorescence ratio of Syto9/PI-stained cells cultured in either low osmolarity TSB medium (left y-axis) or high osmolarity TSB + 20% sucrose broth (Right y-axis). Error bars indicate the standard deviation (*n* = 3).

This study is based on the unsuspected discovery that the *S. minutiscleroticus* cells remained viable in the high osmolarity growth medium for 1 year and 6 months (Figure 1A) until contamination aborted the experiment. The high osmolarity growth medium, TSB + YEME (see materials and methods), used in this study for culturing the bacteria is normally prescribed for dispersed growth and preparation of protoplasts. This medium contains 1X each of TSB and YEME (17% sucrose). YE and ME components have no role in viability. They can be replaced by 20% sucrose (0.584 M), the non-permeable, non-metabolizable (Figure 1B), and non-ionic osmolyte without affecting growth rate. Sucrose, as in YEME only medium, at 34% (1 M) concentration was, however, growth inhibitory. Moreover, TSB + sucrose was reconstituted in these studies by adding filter-sterilized 2X sucrose to 2X autoclaved TSB in 1:1 proportion, although, initially, 20% sucrose was added to the TSB medium, and the two were autoclaved together. Each of the two media supported viability to the same extent (Table 1). We have used in all our experiments TSB added with filter-sterilized sucrose.

**Table 1:** Effect of varying amounts of sucrose supplement on viability (CFU) and protein content of the biomass.

S. No.	Amount of sucrose (gm/100 mL)	Protein concentration (mg/ml)	Viability (number of days)
1	0	1.05	7 days
2	0.1%	0.98	10 days
3	0.2%	1.14	15 days
4	0.4%	1.02	16 days
5	1%	1.09	16 days
6	2%	1.17	18 days
7	5%	1.27	30 days
8	10%	1.25	120–180 days
9	20%	1.28	>365 days

The limited period of enhanced viability (about 2–2.5 months) by metabolizable, non-ionic solutes such as 20% glucose and 20% mannitol (data not shown) aligned well with high amount of nutrients causing effects on LTSP survival of *E coli* (Kram and Finkel, 2015). Ionic solutes, such as sodium chloride, sodium acetate, and potassium acetate, were growth inhibitory between 0.2 M and 0.4 M concentration and ineffective in promoting viability at lesser concentrations (data not shown).

The enhanced conditional viability in LTSP is thus limited to cells cultivated in a high osmotic environment. We presume the MII stage of mycelia persists all through the non-growing state (LTSP) of cells in high osmolar condition on the basis that the MII stage is reached at approximately 100 h (4 days) of growth, likely defining the stationary phase (Dissel et al., 2014; Manteca et al., 2008). The most remarkable effect of sucrose on viability mandates teasing its function as an osmolyte and/or as a source of energy, which was addressed as discussed in the following.

- While *S. minutiscleroticus* does not utilize sucrose as the sole carbon source (Figure 1B) [DSM40301.pdf (dsmz.de)], ~10% of sucrose is hydrolyzed during autoclaving at 121°C, for 15 min (Wann et al., 1990; Wann et al., 1997). We indeed find that sucrose provided in the filter-sterilized form cannot support growth. In contrast, autoclaved sucrose partially does (Figure 1B), with growth levels reaching about 25% of that seen in minimal broth with 0.4% glucose (Supplementary Figure S1). Furthermore, cells in low osmolarity TSB broth supplemented with 2% glucose (an equivalent of 10% hydrolysis of 20% sucrose to glucose during autoclaving) showed viability for about 12 days before entering the death phase. Therefore, the pre-existing glucose and fructose present in the TSB + sucrose at the start of the experiment could not have supported viability from 30 days to above 12 months without the concomitant supply of nutrients from either turnover of cells or from utilization of sucrose consequent to its osmotic role, a proposal we lend credence to, as discussed below.
- The osmotic effect of the sucrose supplement (both autoclaved and filter sterilized) at varying concentrations was correlated with viability and the protein content (as a proxy to several cells) at the end of the growth (Table 1). Regardless of how the sucrose supplement was provided, our findings suggested that the nutrient content of TSB ± sucrose supports equivalent biomass (Table 1). Interestingly, the viability of the culture appears to positively correlate with the osmotic strength of the medium (Table 1), perceived possibly in terms of the strength of the signal and its effect on gene transcription. The enhancement in viability effected by 0.1–2% sucrose is approximately 10–20 days, whereas sucrose at 5, 10, and 20% concentration increased longevity by 1 month, 4–6 months, and > 12 months, respectively.
- Additionally, we also performed manipulations and found that the cells cultured in TSB for 2, 3, 4, and 5 days, when transferred to 20% sucrose solution lacking other nutrients, lost viability in about 15 days. However, if they were also grown in TSB with 20% sucrose, their viability persisted for as long as 3 months, the duration of the experiment. Similarly, cells grown for 30–90 days in high osmolarity TSB + sucrose, when transferred to 2% sucrose (without nutrients) lost viability in ~25 days. This suggests that persistent exposure to high osmolarity is required to preserve cell viability, possibly through continual expression of transcriptome/proteome characteristics of growth under that condition. The results above corroborate the osmotic effect of sucrose to be primarily important for the viability of LTSP cells. Nonetheless, suggestive evidence for slow/poor sucrose utilization as an energy source for sustaining viability in high osmolar growth conditions is presented in the following.
- The cell-free extract prepared from 2 days old low osmolarity TSB culture did not exhibit measurable hydrolysis of sucrose (as measured by GOD–POD method of glucose estimation) when assayed with 1% sucrose as the substrate overnight at 37°C (Table 2). In contrast, extract of cells cultivated in high osmolarity TSB + sucrose for 10, 30, and 90 days formed glucose through a less efficient, likely nonspecific, enzymatic activity estimated at 0.025 μgm of glucose formed from 1% sucrose/min/mg protein (Table 2). Given this specific activity, 13.5 mg of sucrose is hydrolyzed in a year from 20% sucrose (or 200 mg/mL) provided in the medium. This could potentially provide energy for ~15 years to support viability. The hydrolytic function is expressed through the osmotic induction of gene expression program in cells grown in high osmolarity conditions that are absent in low osmolarity grown cells.

**Table 2:** Non-specific sucrose hydrolyzing activity in cell-free extract of TSB + 20% sucrose-grown cells.

S. No.	Different days of high osmolarity culture	Specific activity (μg of glucose produced from 1% sucrose hydrolysis/mg protein/min)
1	10 days	0.02599
2	20 days	0.03031
3	30 days	0.01867
4	90 days	0.01995
**Different days of low osmolarity culture**		
1	3 days	Un-detectable (same as blank)
2	5 days	Un-detectable (same as blank)

In summary, the viability of cells in the long-term stationary phase is majorly due to the exposure of cells to the osmotic effect of non-ionic, impermeable solutes. Sucrose presents an interesting case. Although non-fermentable (Figure 1B), it strikingly supports culturability for the longest time in the lab setting. This unique role of sucrose stems from its initial function as an osmolyte inducing gene expression changes that includes a non-specific and poor hydrolytic function for its utilization as an energy source.

### The long-term viable stationary phase cells are mycelial cells

*Streptomyces* genera consist of soil-dwelling, spore-forming species and the spore form of the differentiated cell survives extremes of environmental challenges. We, therefore, investigated whether the spores produced in submerged cultures of certain *Streptomyces* strains (Dissel et al., 2014) contribute to the survival of cells cultivated in TSB + sucrose medium. The fact that the viable cells (15 days to 90 days) were sensitive to being killed by heat (20 min at 55°C) (Figures 1Cb,c) and by sonication (total time: 1 min 15 s pulse on and 5-s pulse off; 20% amplitude) (Figures 1Ce,f) whereas spores resist these treatments under the same conditions (Figures 1Ca,d), verifies that the LTSP cells are indeed vegetative cells.

### Growth curve of *Streptomyces minutiscleroticus* in low and high osmolarity growth media

The low osmolar TSB and the high osmolar TSB + 20% sucrose broth were each inoculated with ~10^6^/spores/ml and grown at 30°C for the period specified in Figure 1D. The growth curve was constructed using CFU and protein content to demonstrate different stages of growth. Monitoring of growth also included fluorescence ratio measurements of Syto9/PI-stained cells.

**Figure 2:**
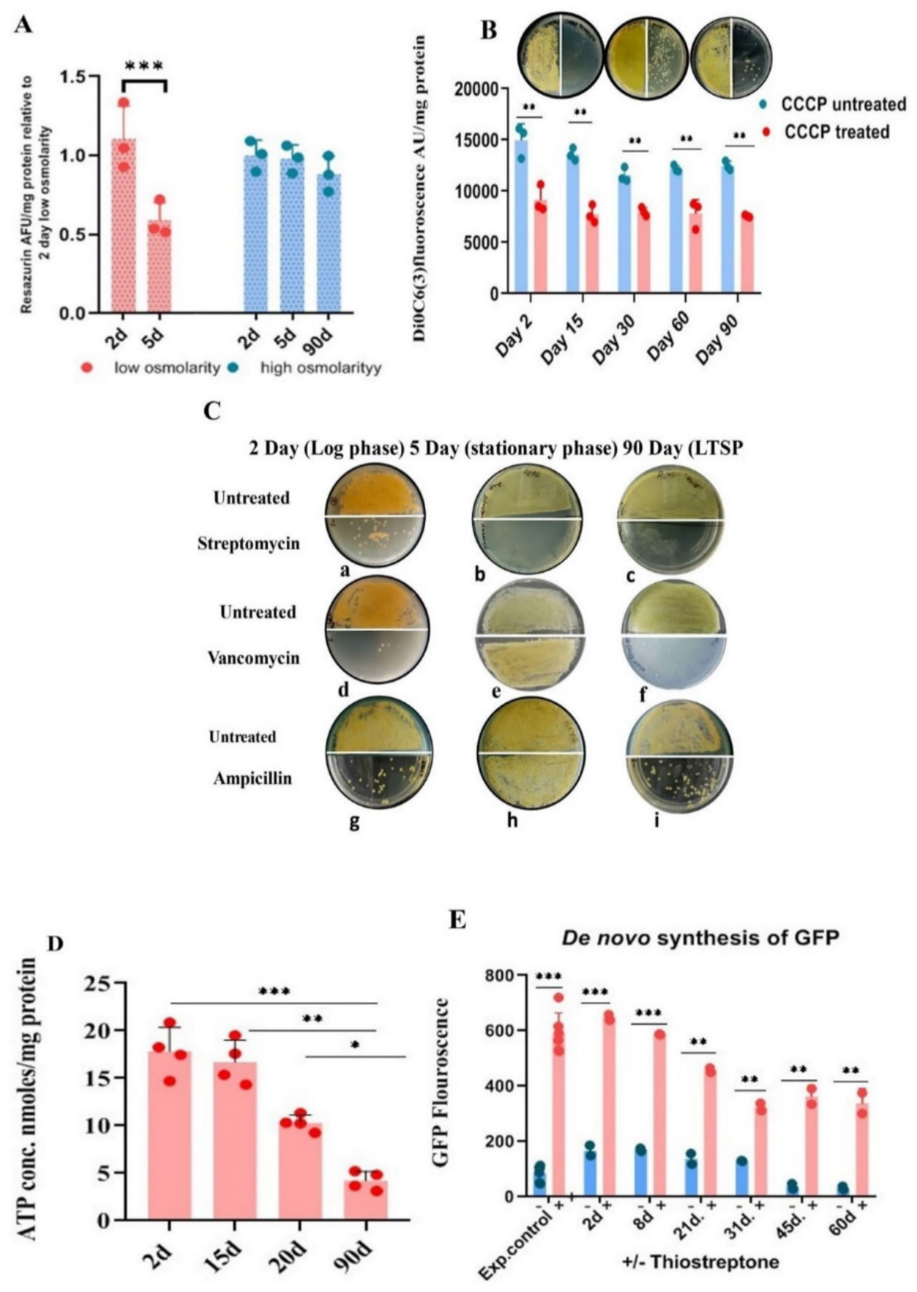
Metabolic status of LTSP cells. **(A)** Metabolic activity measured by reduction of resazurin: Similar to exponentially growing cells, LTSP cells can efficiently reduce blue non-fluorescent dye resazurin to fluorescent resorufin, a measure of the metabolically active status of the cells. Representative data from three independent experiments are shown. **(B)** LTSP cells actively maintain membrane potential: The membrane potential of cells, 2 days through 90 days, can be dissipated by the ionophore CCCP that results in the decrease in the accumulation of membrane potential-sensitive dye DiOC6(3). The effect of treatment on reduction in CFU is evident in plating 50 μL of cells at 10^0^ and at 10^−1^ dilution on the left-and right half of the agar plate, respectively. Representative data from three independent experiments are shown. **(C)** Antibiotic sensitivity profile of LTSP cells: Representative images show that cells from all stages of growth are sensitive to streptomycin (a, b, c). Both LTSP (90 days) (f, i) and exponentially growing cells (2 days) (d, g) are killed by cell-wall synthesis inhibitors, vancomycin, and ampicillin. Stationary phase cells (5 days grown in low osmolarity TSB), on the contrary, are resistant to vancomycin and ampicillin (e, h). **(D)** Determination of ATP concentration in the cells cultured for different days in high osmolar medium to stationary phase: ATP concentration was determined in the cells cultivated in high osmolar medium for different days (from 2 to 90 days) using the kit from Invitrogen (A22066). Representative data from four independent experiments are shown. **(E)** The *de novo* synthesis of GFP in LTSP cells grown for different days in high osmolarity medium: *de novo* GFP synthesis was measured in cells grown for different days in high osmolarity broth by fluorescence spectroscopy. Induction of GFP was carried out by the addition of 50 μg/mL of thiostrepton to cells drawn on different days, measured after 48 h of treatment with the inducer and compared with untreated cells and the control (exponential cells control) cells cultured in the presence of thiostrepton for 2–3 days. Representative data from two independent experiments are shown.

A typical bacterial growth curve is evident when *S. minutiscleroticus* cells grow in low osmolarity TSB medium using CFU count as a measure of growth, consisting of a lag-, exponential-, stationary-, and a death phase (Figure 1D). It is noteworthy that distinct growth phases are apparent when using CFU or OD_540_ as growth indicators, even in *Streptomyces* (Zacchetti et al., 2018; Li et al., 2015; Chua et al., 2013). The importance of CFU is evident in the result that the protein content (or dry mass), which is commonly used metrics to quantify growth, would not decrease during the death phase, as is evident from the constant values of the former estimated from 55 to 180 h, a period during which the CFU count decrease drastically in the growth curve. Interestingly, for the cells grown in high osmolar TSB + 20% sucrose medium, the curves representing protein content and viable count overlap for this experiment (3 months) (Figure 1D), suggesting an absence of the death phase. Notwithstanding the absence of growth difference between 5 days cells from low osmolar TSB with that of the cells cultured in the presence of sucrose, the physiological state of each cell type is conspicuously different (see Figures 2A,C).

In this 3-month experiment, we monitored the Syto9/PI staining profile (Figure 1E) of the cells at different stages of growth by measuring the fluorescence of each dye-stained cells by spectrofluorometer. Exponential phase (2–3 days), cells cultivated in low osmolar TSB medium predominantly stained green with a small number staining red; however, by the early stationary phase (5 days), the proportion of green/red stained cells was approximately equal. As anticipated, cells in the late stationary phase stained mostly red (Figures 1A,E), signifying cell death. Notably, cells cultivated in high osmolarity TSB with 20% sucrose maintained a consistent Syto9/PI ratio even in the advanced starvation phase, with a predominance of green-stained cells over red PI-stained cells at nearly all observed times (Figure 1E). This is manifest in the apparent lack of death phase in the growth curve of cells cultured in high osmolarity TSB + sucrose, which we attribute majorly to the slow turnover of sucrose (Table 2) consequent to induction of gene expression program under the osmotic effect of sucrose. The genetic mechanism of viability operating for cells in high osmolarity overrides the *en masse* death of cells in a low osmolar medium that may include PCD and starvation-induced cell death beyond 7–10 days of being in the stationary phase. We also verified cell viability on different days by examining Syto9/PI-stained cells by confocal imaging (Figure 1A).

### Metabolic status of the LTSP cells

#### Assessment of the metabolic state of LTSP cells using resazurin reduction assay

Maintenance of PMF by the respiratory activity involving metabolic flux through the TCA cycle and electron transport chain is central to cellular functions such as transporting biosynthetic substrates into the cell, functioning of low-and high-affinity transporters and ATP synthesis, even for cells in starvation phase (Bergkessel et al., 2016; Nystrom and Gustavsson, 1998; Matin, 1991; Schneider and Gourse, 2004). Parameters such as respiration, PMF, and ATP concentration, are indicative of the cells’ metabolic status.

Actively respiring cells reduce the non-fluorescent blue resazurin to highly fluorescent purple-colored resorufin, which can be quantified spectrophotometrically or fluorometrically. We evaluated the metabolic status of bacteria cultured in both low osmolar TSB and high osmolar TSB with 20% sucrose during exponential, stationary, and LTSP. Significantly, the metabolic activity level in cells cultured in high osmolar TSB with sucrose for 90 days was comparable to that of cells in the exponential growth phase (2–3 days). In contrast, the cells grown for 5 days to stationary phase in low osmolar TSB medium reduced resazurin less effectively, indicating their lowered metabolic potential in comparison to both exponentially growing (2–3 days old) cells or 90 days LTSP cells (Figure 2A).

#### LTSP cells maintain active membrane potential

The respiratory activity of the cells would translate into maintenance of PMF optimum for metabolic functions. The role of PMF in cell survival in LTSP was assayed using CCCP and uptake of aminoglycosides antibiotics which are PMF dependent. The proton ionophore, CCCP at 100 μg/mL concentration led to a reduction in the CFU count by more than 10-fold when LTSP-and cells in the exponential stage of growth were treated for 48 h at 30°C (Figure 2B). The dissipation of PMF by CCCP along with the reduction in the accumulation of DiOC6(3)—a dye that measures membrane potential—in comparison to untreated control cells (Figure 2B), elegantly demonstrates that the metabolically active, respiring LTSP cells maintain PMF to remain viable in long-drawn nongrowing state.

### Antibiotic sensitivity profile of the LTSP cells

Antibiotic action of aminoglycosides can be potentiated by PMF against slow-growing nondividing cells, including persisters, by promoting its uptake in cells in which it is otherwise ineffective (Allison et al., 2011; Taber et al., 1987; Davis, 1987; Bruni and Kralj, 2020), a reason for the failure of the antibiotic regimen. One of the interesting assays for measuring the metabolic potential of the LTSP cells is based on their growth inhibition by PMF-dependent uptake of aminoglycoside antibiotics. To evaluate this, LTSP cell sensitivity to antibiotics was compared with cells in exponential growth (cultured for 2 days in low or high osmolarity TSB with sucrose) and stationary phase (cultured for 5 days in low osmolarity TSB). Cells were treated with 50 μg/mL of streptomycin and vancomycin. Streptomycin completely eliminated the viability of all cell types when treated for 48 h in either the spent medium or in 10.3% sucrose solution at 30°C (Figures 2Ca–c), consistent with the need for ongoing protein synthesis to maintain viability across all phases of growth (Gray et al., 2019; Yin et al., 2019; Gefen et al., 2014; Reeve et al., 1984; Fuge et al., 1994). The killing of LTSP cells by vancomycin and ampicillin at 50 μg/mL, just as the cells in the exponential stage of growth (Figures 2Cd,f,g,i) was extremely surprising, given that the cells were in stationary phase for more than 10 days of growth (Hazan et al., 2021). This contrasted with the 5–6 days old cells grown in low osmolar TSB medium to stationary phase being sensitive to streptomycin (Figure 2Cb) but resistant to vancomycin and ampicillin (Figures 2Ce,h). Typically, stationary phase/dormant cells are resistant to antibiotics that target cell-wall/cell division, distinguishing them from actively dividing cells (Gray et al., 2019; Yin et al., 2019).

### Intracellular ATP levels decrease in LTSP cells between 15 and 21 days

Although cessation of growth in the stationary phase is marked by a decrease in macromolecular synthesis and reduction in intracellular ATP levels (Dworkin and Harwood, 2022; Schneider and Gourse, 2004), oligotrophic bacteria, including *S. minutiscleroticus*, synthesize ATP by utilizing nutrients from the environment for the maintenance of cellular functions. We compared the ATP content of the cells in the exponential phase (2 days), stationary phase (~15 days), late stationary phase (21 days), and in LTSP cells (90 days) (using the ATP determination kit A22066, from Molecular Probes, Invitrogen). The ATP concentration in 2 days cells was equivalent to that in 15 days; however, the ATP levels declined in the cells between 15 and 21 days and continued to decrease further up to 90 days (Figure 2D). Given the consistent growth rate in both low and high osmolar TSB media and the fact that stationary phase is reached in about 5–6 days in low osmolar TSB (Figure 1D), it is likely that cells in high osmolar TSB + sucrose medium enter stationary phase around the same time. Cells are considered to be in a stationary phase for up to 10 days, after which they enter a deep stationary phase (Hazan et al., 2021). The decrease in ATP levels in LTSP cells is in accordance with its requirement for maintenance function.

### Demonstration of ongoing protein synthesis in LTSP cells

Protein synthesis in the stationary phase (Gefen et al., 2014) is essential for the maintenance of cell viability across various model organisms of senescence (Reeve et al., 1984; Fuge et al., 1994). Accordingly, inhibition of protein synthesis reduces lifespan (Yin et al., 2019; Jaishankar and Srivastava, 2017). Quantifying production of GFP from a thiostrepton-inducible promoter in the vector pIJ8655 (Kieser et al., 2000) integrated into the genome of *S. minutiscleroticus* at the *attB* site (JP5) served to evaluate the *de novo* protein synthesis in the LTSP cells. Cells drawn over a span of 2–60 days were exposed to thiostrepton inducer for 48 h, and their GFP fluorescence was compared with that of cells cultured in the presence of thiostrepton for the same duration achieving full GFP induction. Remarkably, the normalized GFP fluorescence in the cells cultured for an extended period of time in the stationary phase is about ~60% of its maximum inducible value (Figure 2E), demonstrating that the cells in deep stationary phase are capable of protein synthesis, are metabolically active and that viability is obligatorily dependent on protein synthesis.

In conclusion, the threshold of ongoing metabolic activity, measured by respiratory potential, maintenance of PMF, ATP concentration, and capacity to synthesize proteins, is sufficiently high to permit protein-and cell-wall-synthesis inhibitors to effectively prevent CFU formation. This activity is significantly greater than that of 5-day-old stationary phase cells grown in low osmolarity TSB medium. The critical role of ongoing protein synthesis in maintaining cell viability in all phases of growth is apparent from the sensitivity of these cells to protein synthesis inhibitors.

### Limited transcriptome analysis of LTSP cells in deep stationary phase

The evidence from the previous section clearly indicated that the longevity of *S. minutiscleroticus* cells involves elicitation of the genetic program in response to the osmotic stress that can be interpreted particularly by the upregulation of transcriptome/proteome expressed under that condition. We arbitrarily chose 90 days of growth as representative of the LTSP state, also for the technical reason that it was difficult to handle dispersed cells from the highly viscous sucrose medium beyond this stage, however, we do not rule out the possibility of change in structure of the transcriptome over different days in deep stationary phase. We carried out limited transcriptome analysis of one replicate culture of stationary phase cells grown in TSB for 5 days and of cells grown for 90 days in TSB + sucrose. This would help us to select genes from differential gene expression analysis for investigating their role in LTSP. We observed that the log2 value of overexpressed genes ranged from 3 to 12 compared to the narrower range (log2, 2–3) of downregulated genes. This is in accordance with the exclusivity of gene expression under high osmolarity, stationary phase.

A cursory glance at the analysis of the transcriptome data (Supplementary Table S3), although limited for quality in terms of number of replicates sequenced, prominently reveals a set of upregulated genes that appear overtly compatible with the adaptive changes required for survival in deep stationary phase.

Out of 734 transcripts upregulated in 90 days LTSP cells (TSB + sucrose) in relation to the 5 days cells (TSB), ~50% (370) are those for hypothetical and putative proteins of unknown function. From the 364 remaining RNA transcripts (after disregarding transcripts whose log2 values were less than 2), 73 transcripts included almost all ribosomal proteins and translation factors; 23 for proteostasis (chaperones, proteasome, and proteases), 20 transcriptional factors, 16 ATP generation functions; 21 transporters; 6 antioxidant functions; 7 for protein secretion; 12 for DNA repair, integrity and cell division; 53 for proteins involved in altered metabolic function and 8 for macromolecular degradation (Supplementary Table S3).

**Figure 3:**
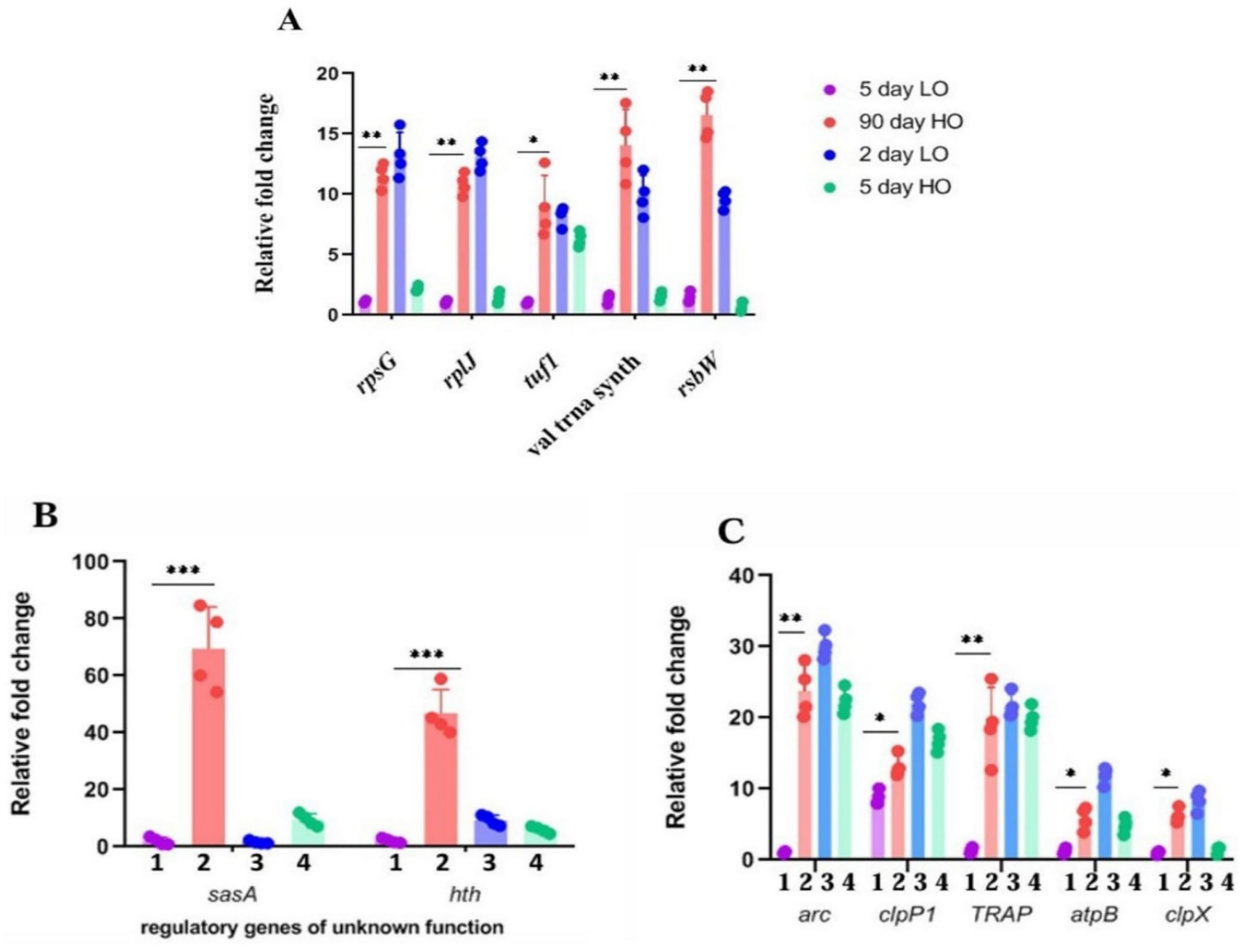
**(A–C)** Validation by quantitative RT-PCR of candidate genes overexpressed in transcriptomic studies of LTSP cells: Relative fold change in the expression of candidate genes from the RNA samples prepared from cells cultured for 5 days in TSB (1), 90 days in TSB + sucrose (2), 2 days in TSB (3), 5 days TSB + sucrose (4) media. The expression of the target gene was normalized to the expression of the housekeeping gene *pfk2*. Error bars show the standard deviation (SD) of biological replicates (*n* = 4).

From the genes that are significantly overexpressed, we chose genes representative of clusters for different functions for their confirmation by quantitative real-time PCR. We found that about 90% of the 19 genes selected for their high expression on the basis of log2 value > 5 could be validated by either RT-PCR (Figures 3A–C) or by enzyme assays (Supplementary Table S4).

Genes with high expression levels from the categories of transcriptional regulators, signal sensor/transducer proteins, antioxidant defense proteins, and metabolic proteins were also analyzed for their role in viability in LTSP (Supplementary Table S5). The rationale for targeting these genes is their presumptuous role in survival in stationary phase and anoxic conditions (Jeong et al., 2018; Hartman et al., 2014; Hampshire et al., 2004; Kwong et al., 2017) in other bacterial systems. We used both strategies as appropriate, namely, (i) examining the effect of its overexpression (by cloning the gene under a constitutive, strong promoter), although sometimes, the latter may also be associated with a new phenotype (Prelich, 2012). (ii) For the valid mutation-phenotype correlation in the knock-out (KO) mutant, we ensured that the gene chosen for mutagenesis was not followed by a downstream gene in an operon format, which incidentally was the case in our studies so that the function of the disrupted gene is directly responsible for the phenotype of viability. A null mutation in several of the genes did not lead to loss of viability in LTSP cells (Supplementary Table S5). The gene functions that affected viability in LTSP are described in the section below.

### Mutations that impact the viability of LTSP cells

#### Involvement of putative histidine kinases in LTSP

Bacterial response to environmental stimuli often involves two-component sensor/responder protein modules (West and Stock, 2001; Jung et al., 2012). Their direct role in sensing osmotic stress and nutritional deficiencies is well known. It is reasonable to assume that the adaptation to these stresses may contain a component of transcriptional readjustments involving a sensory/responder two-component system. We searched for protein kinases/transcriptional regulators that are upregulated in the differential analysis of the transcriptome of cells in LTSP.

**Figure 4:**
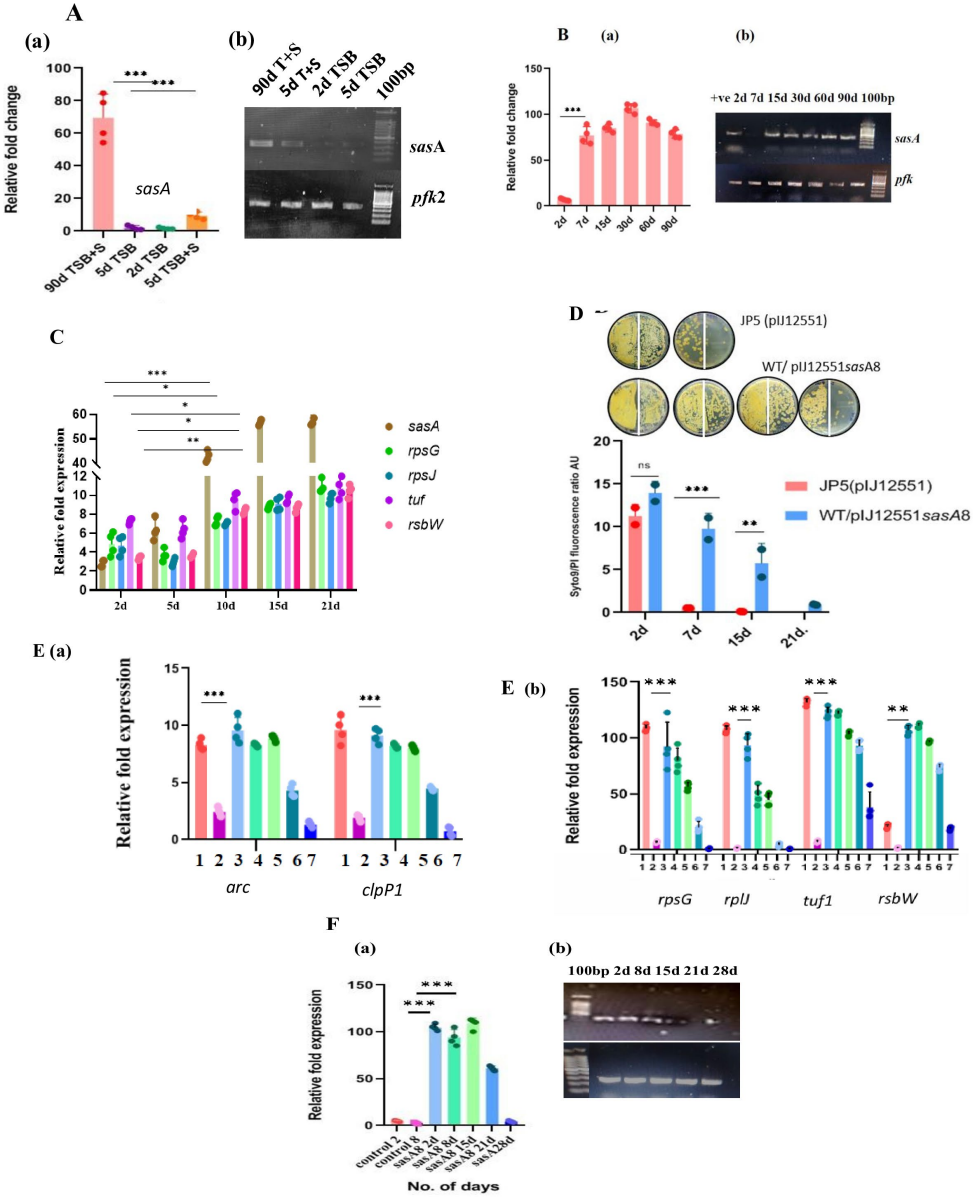
Expression analysis of *sasA8* and other related transcripts **(A)** *sasA8* expression is limited to cells grown in high osmolarity (TSB + 20% sucrose): The *sasA8* transcript amounts measured by quantitative RT-PCR is very high in cells grown in high osmolarity to late stationary phase (90 d TSB + S); it is undetectably low in cells grown to exponential phase in high osmolarity medium (2 d TSB + S) and in cells grown in low osmolarity to either exponential (2 d TSB) or stationary phase (5 d TSB) (a: fold-change in real-time PCR, b - representative electrophoresis gel picture of the PCR products). The expression of the target gene was normalized to the expression of the housekeeping gene *pfk2*. Error bars show the SD of biological replicates (*n* = 4). **(B)** Sustained expression of *sasA8* from 2 days through 90 days: *sasA8* transcript amounts were quantitated from the same amount of RNA isolated from cells grown in a medium of high osmolarity (TSB + sucrose) for different days (a: fold-change in real-time PCR, b: representative electrophoresis gel picture of the PCR products). Expression of the target gene was normalized to the expression of the housekeeping gene *pfk2*. Error bars show SD of biological replicates (*n* = 4); **(C)**. Expression levels of *sasA8* and ribosomal proteins/translation factors are coincident: *sasA8*, *rplJ*, *rpsG*, *tuf*, and *rsbW* transcripts amounts were quantitated from the same amount of RNA isolated from cells grown in a medium of high osmolarity (TSB + sucrose) for different days. Error bars show SD of biological replicates (*n* = 4); **(D)** Viability of cells overexpressing *sasA8* constitutively is enhanced in low osmolarity growth medium: *sasA8* overexpression from strong constitutive promoter, ermE*, enhances viability of cells to more than 15 days to 21 days as against the complete loss of culturability of the control cells by 7 days of growth in medium of low osmolarity (TSB). Viability was measured by both CFU count and Syto9/PI ratio. 50 μL of cells were plated at 10^0^ and at 10^−1^ dilution on the left-and on the right half of the agar plate, respectively. Representative data from three independent experiments are shown. **(E)** (a, b) Overexpression of *sasA8* in cells grown in low osmolarity medium (TSB) increases expression of a few candidate genes characteristically expressed in LTSP cells: Candidate genes that were chosen on the basis of their increased expression in 90 days LTSP cells were analyzed by quantitative RT-PCR in cells overexpressing *sasA8*. 1, day 2 control (WT/pSET152); 2, day 7 control (WT/pSET152); 3, day2 WT/pVM4*sasA8*^+^; 4, day 7 WT/pVM4*sasA8*^+^; 5, day 15 WT/pVM4*sasA8*^+^; 6, day 21 WT/pVM4*sasA8*^+^; 7, day 28 WT/pVM4*sasA8*^+^. The expression of the target gene was normalized to the expression of the housekeeping gene *pfk2*. Error bars show the SD of biological replicates (*n* = 4). **(F)** *sasA8* transcript overexpression is limited to about 15 days: (a) *sasA8* transcript overexpression is tenable till 15 days of growth in low osmolarity TSB broth. The levels are very high in 2 days, 7 days, and 15 days cells, and declines from 21 days onward. The *sasA8* transcript is undetectable in control cells (JP2) (in day 2 and day 5 cells from low osmolarity broth). (b) Representative electrophoresis gel picture of the PCR products. The expression of the target gene was normalized to the expression of the housekeeping gene *pfk2*. Error bars show the SD of biological replicates (*n* = 4).

One predicted histidine kinase gene, *sasA8*, was highly upregulated (log2-13.2) in the comparative transcriptome analysis. We reassessed the expression of this gene by quantitative RT-PCR (Figures 4Aa,b) and found that its expression was remarkably low in cells in low osmolar exponential growth, stationary phase and also in cells in exponential phase cultivated in high osmolarity medium, whereas the expression was at its maximum from the seventh day of growth in high osmolar medium and continue to remain high through 15-, 30-, 60-, and 90 days (Figures 4Ba,b). Furthermore, an interesting correlation was apparent in the simultaneous expression of *sasA* and ribosomal proteins/translation factors suggesting the *sasA*’s function in regulating that portion of the stationary phase proteome. For example, *sasA* transcript levels conspicuously increase from 5 days through 90 days in cells grown in TSB + sucrose (Figures 4Ba,b), and so is an expression of ribosomal proteins and translation factors (Figure 4C).

The unique pattern of *sasA8* gene expression appears to suggest the importance of the gene’s function for survival in LTSP. *sasA8* gene was PCR amplified using primers (SasA8 Left and SasA8 Right, Supplementary Table S2), cloned under the ermE* promoter in the vector pIJ12551 (Supplementary Table S1) and introduced into wild type *Streptomyces* strain. The control JP5 (WT/pIJ12551) and transformants (WT/pVM3*sasA8*) were each grown in low osmolar TSB broth, and the viability was monitored by plating for CFU on R2YE agar and by staining with Syto9/PI (Figure 4D). The results convincingly demonstrated that constitutive expression of *sasA8* extended the life span of the stationary phase cells grown in low osmolarity TSB. The cells on 15 days (until ~21 days) stained green, and the CFU count was the same as on the second day, whereas the viability of the empty vector control transformants was completely lost in about 7 days (Figure 4D). Significantly, not only is the viability enhanced, the characteristic pattern of representative genes’ relative fold expression in stationary phase from the categories of ClpPX protease (*clpP1*), proteasomes (*arc*), translation factor (*tuf*), ribosome proteins (*rplJ*, *rpsG*), and *rsbW* (antisigma factor) were also upregulated (Figures 4Ea,b), similar to their enhanced expression in the >5 days to 90 days old cells (Figures 3A–C, 4C). This pattern is provocatively suggestive of the importance of the phosphoproteome in gene expression in nutrient-starved conditions and that higher levels of ribosomal proteins are required for new protein synthesis and its turnover by proteolytic activity for LTSP survival. The limited enhancement of viability of cells overexpressing *sasA8* under the ermE* promoter is in contrast to its continual expression in 3-month-old cells, and more. However, the result is not unexpected. Sustained expression of *sasA8* under the stress of high osmolarity and the stationary phase is most likely to involve transcriptional factors modifying RNA polymerase specificity for promoters transcribing genes under the stress conditions, whereas transcriptional activity of highly active constitutive promoter is expected to be untenable under nutrient deprivation conditions. Indeed, *sasA8* expression dissipated by 21–28 days when compared to that in 3, 7, and 15-day cells (Figures 4Fa,b). Since *sasA8* expression under its native conditions is expected to be factor dependent, expression under its’ own promoter in multicopy is unlikely to enhance the viability of cells grown in low osmolarity TSB. Given the expression pattern of the *sasA8* gene, the gene function was assumed to be inessential for viability in the exponential phase of growth. The chromosomal copy of *sasA8* was disrupted by homologous recombination-mediated integration of the gene disruption plasmid (described in materials and methods). Surprisingly, the *sasA8* mutation did not affect the viability of the mutant in the stationary phase in media of high osmolarity, suggesting the existence of overlapping multiple kinase functions. Indeed, the chromosome of *S. minutiscleroticus* contains gene sequences for about nine histidine kinases, the *sasA8* targeted for the gene disruption is the strongly expressed gene in cells grown to stationary phase at high osmolarity (Figure 4A).

### Mutation in *clpX* causes loss of viability of LTSP cells

**Figure 5:**
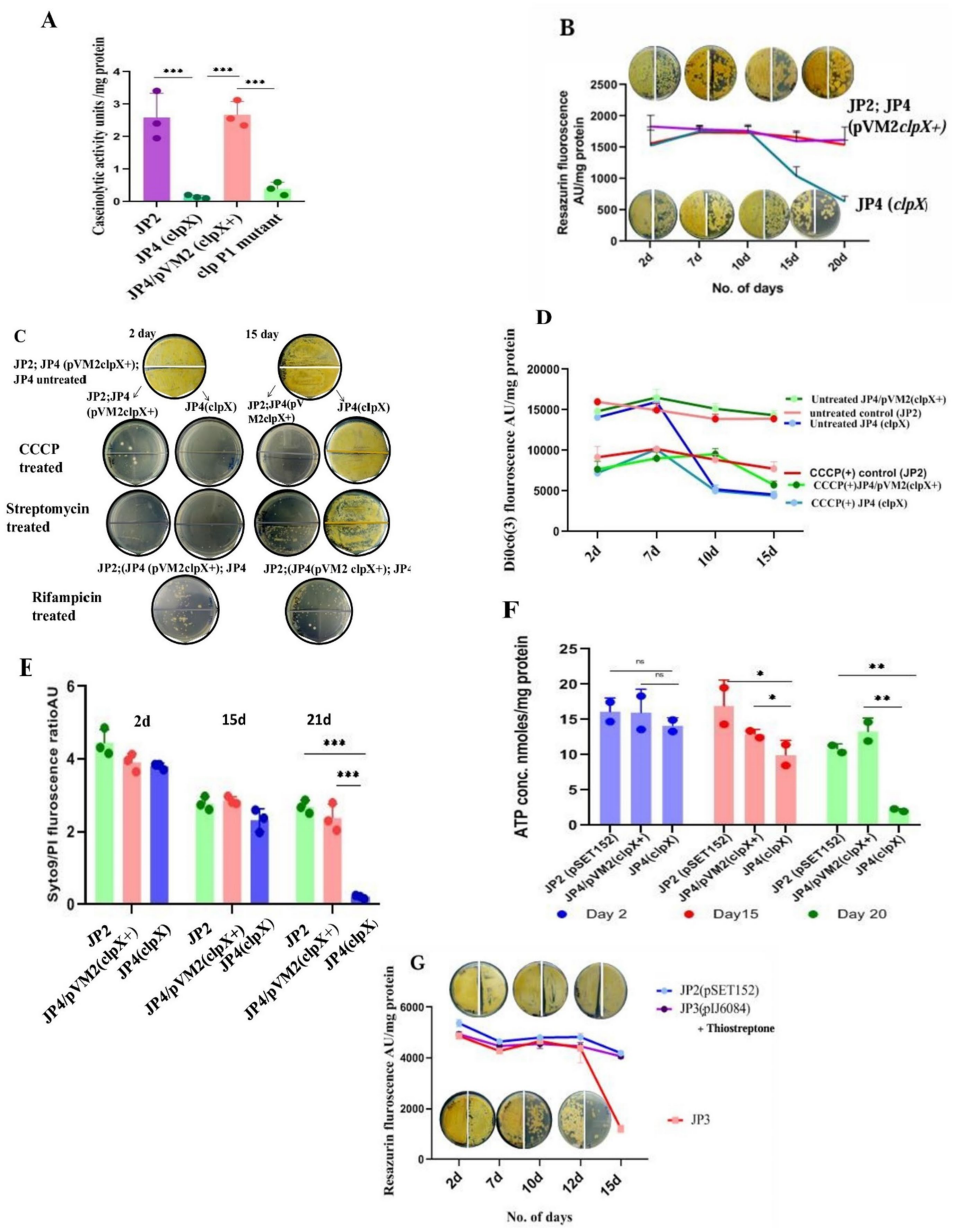
Mutations compromising the viability of LTSP cells. **(A)** Loss of caseinolytic activity of the *clp* mutants: Caseinolytic activity (measured by a decrease in the absorbance of casein at 600 nm) of *clpX* (JP4) and *clpP1* mutants was drastically reduced in an *in vitro* assay using cell-free extract and 1% casein solution. Representative data from three independent experiments are shown. **(B)** Metabolic activity of *clpX* (JP4) mutant as measured by reduction of resazurin and its correlation with viability (measured by CFU): Reduction of resazurin to resorufin by the cells of *clpX*, WT (JP2) and complementing strain, JP4/pVM2 *clpX*+ cultured in media of high osmolarity TSB + sucrose for different days. CFU was enumerated at the corresponding time points. Note that the CFU count on 15 days was the same as that of the earlier time points, even though the resazurin fluorescence decreased significantly. 50 μL of cells on day 2, day 7, day 10, and day 20 were plated at 10^0^ and at 10^−1^ dilution on the left and the right half of the agar plate, respectively. Representative data from three independent experiments are shown. **(C)** The state of PMF being refractory to dissipation by CCCP in *clpX* mutant is associated with resistance to growth inhibition by CCCP and streptomycin: Cells of JP4 (*clpX*), WT (JP2) and complementing strain (JP4/pVM2 *clpX*^+^) grown in media of high osmolarity TSB + sucrose for 2 days and 15 days were treated with CCCP (at 30 μM) and streptomycin (at 100 μg/mL) for 48 h and plated for CFU count. Note the *clpX* cells are unresponsive to the inhibitory effects of CCCP and streptomycin in contrast to the mutant (JP4) cells on 2 days or the control [JP2; JP4 (pVM2 *clpX*^+^)] cells. Rifampicin is inhibitory for growth for all cell types irrespective of their day of growth. The representative images are shown. **(D)** The state of PMF being refractory to dissipation by CCCP in *clpX* mutant is evident in lowered values of fluorescence of membrane potential measuring dye DiOC6(3). Changes in the membrane potential of the *clpX* (JP4) mutant in comparison with the cells of wild type and complementing strain JP4/pVM2 (*clpX*^+^) cultured in high osmolarity TSB + sucrose medium for different days demonstrates a decline in the PMF that remains unchanged even after treatment with CCCP. Unlike dissipation of membrane potential in all other types of cells grown for different days, including *clpX* mutant on 2 days and 7 days, these cells from 10 days onward up to 18 days are refractory to dissipation of PMF by CCCP. Representative data from three independent experiments are shown. **(E)** Syto/PI staining of the *clpX* mutant cells for assessing membrane integrity: Cells of JP4 (*clpX*), WT (JP2), and complementing strain (JP4/pVM2 *clpX*+) grown in media of high osmolarity for 2 days, 15 days, and 21 days were stained with Syto/PI. Representative data from three independent experiments are shown. **(F)** Estimation of ATP concentration in cells of *clpX* mutant grown for different days in high osmolarity medium: ATP concentration was estimated in the cells of JP4 (*clpX*), WT (JP2), and complementing strain (JP4/pVM2 *clpX*+) grown in media of high osmolarity for 2 days, 15 days and 21 days using the ATP estimation kit from Invitrogen. The loss of viability correlated with a drastic decrease in ATP concentration in the *clpX* mutant, JP4. Representative data from two independent experiments are shown. **(G)** Loss of viability of *relA* (JP3) mutant cells grown in high osmolar medium in late stationary phase: Viability, as measured by reduction of resazurin dye and by CFU count, decreases concomitantly in the cells of *relA* mutant (JP3) cultured in high osmolarity TSB + sucrose medium between 15 and 18 days in relation to the control [JP2; JP3 (pIJ6084)]. Approximately 50 μL of cells from day 2, day 10, and day 15 were plated at 10^0^ and at 10^−1^ dilution on the left-and on the right half of the agar plate, respectively. The JP3 (pIJ6084) complementing strain was grown in the presence of 10 μg/mL of thiostrepton. Representative data from two independent experiments are shown.

*clpX* encodes the ATP-dependent chaperone subunit of the ClpP1P2X protease (Hanson and Whiteheart, 2005). It is the third gene in the order *clpP1*, *clpP2*, and *clpX*, and all three are oriented in the same direction on the chromosome of *S. minutiscleroticus*. Given the extent of intergenic DNA at the 5′ end of these genes, it is possible that *clpP2* and *clpX* may define an independent transcription unit (intergenic DNA between *clpP1* and *clpP2* is 154 nucleotides and that between *clpP2* and *clpX* is 46 nucleotides). We opted to mutate *clpX* to prevent the insertion mutation in *clpP1* from exerting a polar effect on the expression of the downstream gene (s), which, incidentally, turned out to be advantageous. Five randomly chosen *clpX* insertion mutants (JP4) were shown to have all lost caseinolytic activity in an *in vitro* assay using cell-free extract (Figure 5A) and 1% casein solution as the substrate, a loss that can be complemented by cloned *clpX*^+^ gene in the plasmid, pVM2 (pIJ10257 *clpX^+^*) (Table 1; Figure 5A). Surprisingly, the viability of the mutant culture grown in TSB + 20% sucrose fell precipitously (~50 fold) (Figure 5B; Supplementary Figure S4) between 20 and 24 days compared with the wild type and the *clpX*^+^ complemented strain (JP4/pVM2 *clpX*^+^) which is viable during the same period. Defective unfolded protein response or accumulation of protein aggregates lessens the lifespan of several organisms (Bednarska et al., 2013; Maisonneuve et al., 2008; Weichart et al., 2003; Reeve et al., 1984). These mutants are expected as well to be heat sensitive at 42°C, as exposure to high temperature leads to accelerated production of denatured proteins and protein aggregates, necessitating chaperones for their refolding and proteases for their effective removal (Weichart et al., 2003). Lack of either of the two functions leads to temperature sensitivity. If the loss of protease activity in the *clpX* mutant is responsible for the gradual loss of its viability, the same is expected of the *clpP1* mutant, given that the ClpP1P2X complex possesses proteostasis function under different stress conditions such as heat-, oxidative stress, and nutrient deprivation (Basta et al., 2020; Hanson and Whiteheart, 2005; Reeve et al., 1984; Frees et al., 2007). Intriguingly, both *clpP1* and *clpX* mutants were resistant to being inhibited for growth at 42°C; however, unlike the *clpX* mutant, the *clpP1* mutant was viable for the duration of the experiment, about 30 days or more, similar to the wild type/*clpX*+ complemented strain (data not shown). The absence of temperature-sensitive growth phenotype of *clpX* and *clpP1* single mutants suggests that the clpXP1P2 protease complex is not involved in the removal of protein aggregates, unlike in other bacterial systems (Basta et al., 2020; Reeve et al., 1984). The presence of ORFs for *clpB*, *clpC*, *clpP*, *clpP3*, *clpR*, and *clpP4* and proteasome pupylation-dependent pathways in the genome of *S. minutiscleroticus* may be involved in the protein turnover. Although caseinolytic activity is lost in both *clpX* and *clpP1* single mutants (Figure 5A), the effect of loss of viability is unique to the former but not observed with the latter. This may be suggestive of the *clpX* protein having additional biological functions, including its role in energy homeostasis, in addition to acting as a chaperone (Fischer et al., 2015; Seo et al., 2016; Cohena et al., 2018; Zhang et al., 2022; Kester et al., 2021) and as an ATPase subunit of ClpXP protease (Hanson and Whiteheart, 2005).

In attempting to investigate the reason for the loss of viability of the *clpX* mutant, we find that the mutation has no overt effect on the growth rate (in TSB/TSB + sucrose) or sporulation (data not shown).

- However, when we measured respiration/metabolic activity for cells of *clpX* mutant, wild type, and *clpX*+ complemented strain (JP4/*p*VM2*clpX*^+^) cultivated in TSB + sucrose medium on different days of the growth, we observed that after about 10 days, for the period between 12 and 18 days, the fluorescence associated with resazurin reduction to resorufin of *clpX* cells decreased by half in comparison to the wild type/complemented strain (Figure 5B). Interestingly, the CFU count did not decrease simultaneously. This is in contrast to the linear correlation between loss of viability of *relA* mutant and reduction in resazurin fluorescence (Figure 5G).
- Importantly, the sucrose-washed cells during this stage were resistant to being killed by the PMF dissipating agent, CCCP (100 μg/mL, 48 h) (Figure 5C), in comparison to the cells of wild type and JP4/pVM2 *clpX*^+^ complemented strain, which drastically lost viability under the same conditions (Figure 5C).
- The uptake and growth inhibition by the aminoglycoside, streptomycin, has been shown to be PMF-dependent in several organisms (Allison et al., 2011; Taber et al., 1987; Davis, 1987; Bruni and Kralj, 2020). Accordingly, killing by 50 μg/mL of streptomycin was limited to the sucrose-washed 2-day cells of *clpX* mutant (JP4) and wild type/complemented strain. Strikingly, *clpX* mutant cells, unlike those of wild type/complemented strain between 12 and 18 days, were resistant to being killed by streptomycin (Figure 5C), reconfirming the reduced PMF as the reason for the loss of streptomycin uptake.

Remarkably, killing by Rifampicin, whose uptake is PMF independent, was not affected by the stage (log- /stationary phase), or day of the growth (second day/12–18th day) of both *clpX* mutant and the wild type/complemented strain (Figure 5C).

- In agreement with the reduction in PMF in the JP4 (*clpX*) mutant between 12 and 18 days, the mutant cells fluoresced less (by almost half) with the membrane potential measuring dye DiOC6(3) (30 mM for 20 min) irrespective of the treatment by CCCP (100 μg/mL) (Figure 5D). In contrast, in the cells of wild type/complemented strain, DiOC6(3) fluorescence was higher in the absence of CCCP than in its presence, clearly indicating that JP4 (*clpX*) mutant cells have compromised respiration and PMF during the 12–18 days period. This result indicates that the reduction in PMF in the *clpX* mutant is at the threshold that is unresponsive to further dissipation by CCCP. However, the compromised membrane potential is reversible, and the cells are viable when fresh nutrients are provided in the medium (Figure 5C), that is, they score viable when plated on R2YE agar, also contain more green fluorescing cells (live) than red (dead) by the Syto 9/PI stain (see the following).
- Although the compromised membrane potential of JP4 (*clpX*) cells is reversible and not reflected in the loss of membrane integrity, the mutant grown up to 21 days and beyond failed to form CFU (Supplementary Figure S4) indicating irreversible loss of some important function defining death. When we measured the membrane integrity of cells of *clpX* mutant, WT, and the complemented strain using Syto9/PI dye, we observed that the cells on the second day, and 15–20 days contained a greater number of Syto9 green staining (viable) cells than the PI-stained dead cells (red). Except on days beyond 21 days, where *clpX* mutant cells stained all red by PI, indicating irreversible loss of membrane integrity and thus loss of CFU formation ability, the WT and the complemented strains were more green than red (Figure 5E). Thus, there is a clear demarcation of the cell stage where reduction in PMF can be reversed as against the cells that enter an irreversible state of loss of viability accompanied by loss of both PMF and membrane integrity.
- Fall in the levels of ATP in the *clpX* mutant 2 days through 21 days (Figure 5F) is severe between 15 days and 21 days, consistent with the reduction in the PMF during this period (Figure 5D).
- All of the above parameters measured for the second-day culture of *clpX* mutant (JP4) were the same as that for wild type/complemented strain, indicating that the cells manifest no inherent defect during the exponential phase of growth (Figure 5B through Figure 5F) and that the defect is evident only in late stationary phase (>10 days).

In summary, the events preceding the loss of CFU formation of *clpX* mutant (JP4) is highly informative and consist of two stages. In the first stage, there is a reduction in metabolic activity between 12 and 18 days, leading to a decrease in membrane potential (Figures 5B,D), but relatively intact membrane integrity (Figure 5E), which is reversible when provided with fresh nutrients. Interestingly, the reduction in PMF is reflected in the resistance of cells to inhibition of growth by CCCP and killing by streptomycin antibiotic (Figure 5C) whose uptake is PMF dependent. Eventually, in the second stage, the continued reduction in PMF and metabolic activity leads to a drastic reduction in ATP levels (Figure 5F) and loss of membrane integrity (Figure 5E), where CFU count decreases by more than 50-fold (Supplementary Figure S4). However, the loss of membrane integrity is not accompanied by lysis of the starving biomass, as in the case of *E. coli*, due to loss of ion homeostasis, and sucrose as a neutral osmolyte may play a positive role in osmoprotection (Schink et al., 2021).

### Loss of viability by knock-out mutation in the (p)ppGpp synthetase encoding gene, *relA*

Expression of genes in response to nutrient limitation is predominantly dependent on their transcriptional activation by nucleotide derivatives, ppGpp and pppGpp, collectively referred to as (p)ppGpp, which are necessary for the survival of bacteria in the non-growing state (Dutta et al., 2019; Mathew et al., 2004; Müller et al., 2012). Given that the LTSP is a stationary phase phenomenon, we determined if the genetics of longevity in LTSP is contingent upon (p)ppGpp synthesis, given substantial genetic evidence in its support. However, there is a large variation in the effect of (p)ppGpp on cellular processes between bacterial species, including influence on secondary metabolism and differentiation. There is also variation in the number of functional genes RSH and Rel among *Streptomyces* strains (Sun et al., 2001; Song et al., 2023). *S. minutiscleroticus* genomic DNA harbors two genes related to (p)ppGpp homeostasis. The *relA/spoT* gene that exhibits more than 90% sequence homology with its counterpart from several *Streptomyces* species was chosen for mutagenesis. Intriguingly, we failed to find the effect of *relA* mutation (JP3) on either sporulation or antibiotic production on several media we tested, such as R2YE, R4, SMMS, and SMA (data not shown). Not surprisingly, and in line with the compromised long-term survival of (p)ppGpp^0^ mutants in stationary phase (Dutta et al., 2019; Mathew et al., 2004; Müller et al., 2012), the effect of *relA* mutation on viability on different days of growth was in consonance with the obligatory role of (p)ppGpp in stationary phase gene expression. Metabolic status and viability of *relA* mutant (JP3) cells cultivated in high osmolarity TSB + sucrose were monitored, respectively, with resazurin and by plating on R2YE. The mutants’ viability matched that of the wild type (JP2) and JP3/pIJ6084 (supplemented with 10 μg/mL of thiostrepton) for up to 15 days. Strikingly, after about ~15 days of being in the extended stationary phase, the CFU count of the mutant (JP3) started declining (~50-fold) (Figure 5G; Supplementary Figure S5). As expected, the CFU loss correlated linearly with the decrease in resazurin reduction, in stark contrast with the *clpX* mutant (Figure 5B), suggesting the mechanism of viability loss is different for the *relA* and *clpX* mutants and that survival in LTSP is (p)ppGpp dependent. Although we did not estimate (p)ppGpp levels in the mutant, we contend that the consistent and robust effect of the mutation on viability did not need supporting evidence to demonstrate a decrease in the (p)ppGpp levels.

## Discussion

Bacterial lifespan is largely decided by both genetics and environment. A recent research study discussing the longevity of certain model bacteria, despite the constraints of batch culture models (Hoehler and Jørgensen, 2013), has provided valuable insights (Gray et al., 2019; Zambrano et al., 1993; Dahouk et al., 2013; Lostroh and Voyles, 2010; Hampshire et al., 2004; Lewenza et al., 2018). These studies have shown that LTSP can be achieved in a laboratory setting after nutrients are completely depleted, highlighted the critical role of low-level nutrient availability in the environment for sustained viability, underscored the significant impact of genetic makeup reflective of lifestyle and environmental niche, and described the physiological state of these cells, including their metabolic capabilities and ATP production under severe energy limitations. The model organism studied encompasses varied lifestyle regimes of facultative species—both spore-forming and non-sporulating, heterotrophs, photoautotrophs, pathogens, and obligate aerobes. Our study with an obligately aerobic, spore-forming, Gram-positive bacterium, *S. minutiscleroticus*, unveils an interesting diversity in the genetic program for the long-term survival of quiescent mycelial cells that defies the starvation-related cell death in the stationary phase. The genetic basis is strongly indicated in the continuance of the quiescent state by merely transferring log/stationary/deep stationary phase cells cultivated in TSB + 20% sucrose to 20% sucrose solution without added nutrients. This contrasts the early loss of viability (between 10 and 15 days) of cells cultivated in low osmolarity TSB medium under identical transfer conditions. The role of sustaining longevity is rendered best and uniquely by sucrose at two levels. Its provision in high concentration in the growth medium is first realized for its osmotic character causing transcriptional induction of gene expression. Consequently, the inclusion of a poor, non-specific sucrose hydrolytic activity in the gene expression program generates ATP for sustaining mycelial cell viability in LTSP for maintenance of the metabolic potential (Figures 2A–C,E). ATP generation by photophosphorylation in growth-arrested *R. palustris* has been shown to prolong survival (Yin et al., 2023) and is in line with the importance of basal level of ATP synthesis in sustaining viability.

It is pertinent to understand how the quiescent cell stage of *S. minutiscleroticus* compares with that of other model systems (Dworkin and Harwood, 2022; Pechter et al., 2017; Yin et al., 2019; Gray et al., 2019; Finkel, 2006; Zambrano et al., 1993; Basta et al., 2020; Yin et al., 2023). A near constancy of Syto9/PI ratio of cells from 2 days to 90 days (Figure 1E) and comparable recovery of CFU count about this time (Figure 1D) is proof that a large number of cells of *S. minutiscleroticus* survive in deep stationary phase, similar to the starved cells of *R. palustris* (Yin et al., 2019). Furthermore, the unique sensitivity of LTSP cells to cell division inhibitors vancomycin/ampicillin, unlike the resistance of stationary phase cells (grown for 5 days in low osmolarity TSB) (Figure 2C) indicates a turnover of the LTSP cells. This is in contrast to a small fraction of cells of *B. subtilis* (Gray et al., 2019), *E. coli* (Finkel, 2006), *P. aeruginosa* (Basta et al., 2020; Mawla et al., 2020), and others (Dahouk et al., 2013; Lostroh and Voyles, 2010; Betts et al., 2002) surviving in LTSP following death of a large majority.

The interpretation of the genetic elements by differential gene expression (DGE) analysis of the transcriptome uncovered a pattern coherent with the probable mechanism of survival of LTSP cells with marked overexpression of almost all ribosomal proteins (Figures 3A, 4C,Eb) and the functions vital for translation (Supplementary Table S3). The excess of unassembled ribosomal proteins may act as a reservoir of free amino acids for new protein synthesis in LTSP. Also evident is the concomitant overexpression of different proteases (Figures 3B,C, 4Ea) and chaperones for catalyzing proper folding of newly synthesized proteins. Peptidoglycan remodeling genes are moderately overexpressed in line with the requirement of these functions for its turnover, accounting for vancomycin and ampicillin sensitivity of LTSP cells (Supplementary Table S3). The DGE analysis also identified three candidate genes whose altered expression affected viability in LTSP.

- The survival of LTSP cells in the starvation phase is expected to be dependent on (p)ppGpp as this is also a signal to couple starvation to changes in gene expression required for viability.

The anticipated effect of a mutation in *relA*, the gene encoding (p)ppGpp synthetase, was evident in the null mutant, also called (p)ppGpp^0^, that exhibited early loss (15–18 days) of viability in the stationary phase (Dutta et al., 2019; Mathew et al., 2004; Müller et al., 2012) (Figure 5G; Supplementary Figure S5), demonstrating the RelA/SpoT being the major (p)ppGpp synthetase in the cell and that survival in both stationary phases (Yin et al., 2019; Reeve et al., 1984) and LTSP is obligatorily dependent on (p)ppGpp mediated gene expression.

- The transcriptome study uncovered the role of *clpX* in stationary phase viability, a role not unexpected as ClpX protease/chaperone is required for homeostasis of proteome structure, also in stationary phase (Maisonneuve et al., 2008; Weichart et al., 2003; Frees et al., 2007). However, given the evidence with *clpP1* mutant, the involvement of *clpX* appears independent of its protease activity, suggesting its unconventional role in sustaining viability. Although the proposal that in *clpP1* mutant, ClpX may interact with other Clp proteases in as yet uncharacterized ways in causing protein turnover is a distinct possibility (Mawla et al., 2020; Hall et al., 2017), unlike the linear correspondence between decline in CFU formation of the *relA* mutant (Supplementary Figure S5), and decrease of metabolic functions measured by reduction of resazurin dye (Figure 5G), the loss of viability of *clpX* mutant is quite unusual and is reminiscent of unique effect of *clpX* on the activity of respiratory enzymes in eukaryotes (Cohena et al., 2018). ClpX has uniquely been shown to interact with cytochrome, TCA cycle enzymes in higher eukaryotes, and causing dysregulation of several proteins of TCA cycle in cyanobacteria (Frees et al., 2007; Fischer et al., 2015; Seo et al., 2016; Cohena et al., 2018; Zhang et al., 2022). Evidence of such interaction is wanted in our system. However, the following observations may provide clues to the potential mechanism of viability loss. Respiration decreases midway through the 21 days of the viability period (on the 10th day) (Figure 5B), coinciding with the loss of PMF at about the same time (Figure 5D). The mutant during this time is also insensitive to killing by aminoglycoside antibiotic, streptomycin, and proton ionophore, CCCP (Figure 5C), functions that are obligatorily dependent on both electrochemical potential and pH gradient. Generation of membrane potential is consequent to optimum metabolic functions producing ATP. Therefore, the perceptible reduction in ATP during the 10–18-day window (Figure 5F) and drastically through a non-growing phase, more strongly in *clpX* mutant than in the wild type, we believe, is suggestive of the direct effect on respiration. However, reduction in membrane potential did not reduce viability and membrane integrity between 10 and 20 days (Figures 5B,E), indicating the reversibility of each of these parameters in the presence of nutrient supplementation. Nonetheless, the continuance of diminished PMF/respiration and severe reduction in ATP concentration (Figures 5D,F) post 21 days, eventually led to irreversible loss of viability (Figure 5B; Supplementary Figure S4) and membrane integrity (Figure 5E).

Our study also invokes sensory kinase, *sasA8*, in the longevity of LTSP cells. Protein kinases play a crucial role in cellular signaling and sensing environmental cues (West and Stock, 2001; Jung et al., 2012). The *sasA8* was chosen for further studies for the characteristic pattern of its gene expression limited to cells in the late stationary phase (Figure 4A), qualifying for its potential role in viability. The onset of the death phase on days 6 and 7 in cells in low osmolar TSB apparently coincides with the beginning of increased SasA8 expression in TSB + sucrose-grown cells (Figure 4C), which is sustained till the period of the experiment (Figures 4Ba,b,C). This may suggest a link between the absence of *sasA8* expression and ensuing death in cells in low osmolar TSB. Strikingly, the upregulation of a subset of genes, including ribosomal protein genes/translation functions (Figure 4C), coincident with enhanced expression of *sasA8* (Figure 4C). The association is also evident in the ectopic expression of *sasA8* from strong promoter ermE*, enhancing the viability of cells cultivated in a low osmolar TSB medium (Figure 4D). This is coupled to an increase in the expression of ribosomal protein genes, proteases *clpP*, and proteasome subunit gene *arc* (Figures 4Ea,b), suggesting the correlation between enhanced longevity and expression of *sasA8* mediated phosphoproteome. The tapering of the strong, constitutive ermE* promoter activity in the late stationary phase (~21 days) (Figure 4F) possibly limits viability, reinforcing the correlation. These findings offer a promising direction for the future research to delve into the impact of genetic modifications on longevity during the stationary phase.

## Data Availability

The datasets presented in this study can be found in online repositories. The name of the repository and accession number can be found at: https://www.ncbi.nlm.nih.gov/geo/, PRJNA1110259.
