# Supplementary material for: Genetics of conditional extended mycelial cell viability of Streptomyces minutiscleroticus in deep starvation phase implicates the involvement of (p)ppGpp, clpX, and a histidine kinase sasA
Source: Front Microbiol. 2024 Nov 14;15:1495007. doi: 10.3389/fmicb.2024.1495007 (PMC11604128; doi:10.3389/fmicb.2024.1495007)
Supplement: Supplementary file 1 [file Supplementary_file_1.docx]

Supplementary Information

**Attempt to realize the genetic program of extended mycelial cell viability of *Streptomyces minutiscleroticus* in deep stationary phase.**

**Vaidehi Chatupale^1^ and Jayashree Pohnerkar^1*^**

Department of Biochemistry, The Maharaja Sayajirao University of Baroda, Vadodara, Gujarat, India, 390002;

**^*^**To whom the correspondence is addressed

Email: jayashreepohnerkar@hotmail.com

**Supplementary Figures**

**Fig. S1: Growth of *S. minutiscleroticus* in defined media supplemented with 0.4% each of glucose, autoclaved sucrose and filter sterilized sucrose.**


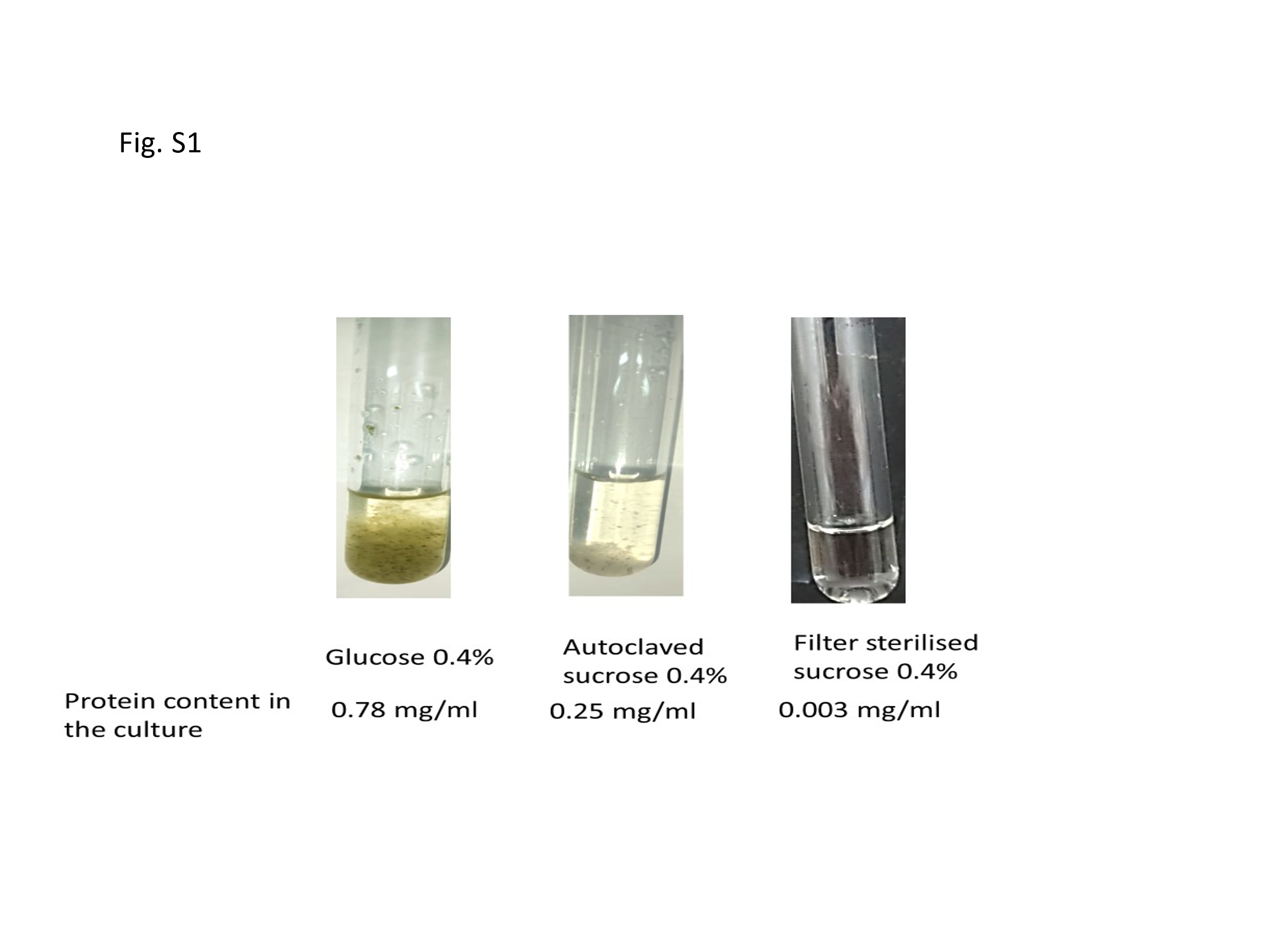


**Figure S2: Stationary phase cells grown in high osmolarity medium produce extracellular diffusible dark brown pigment, possibly melanin.**


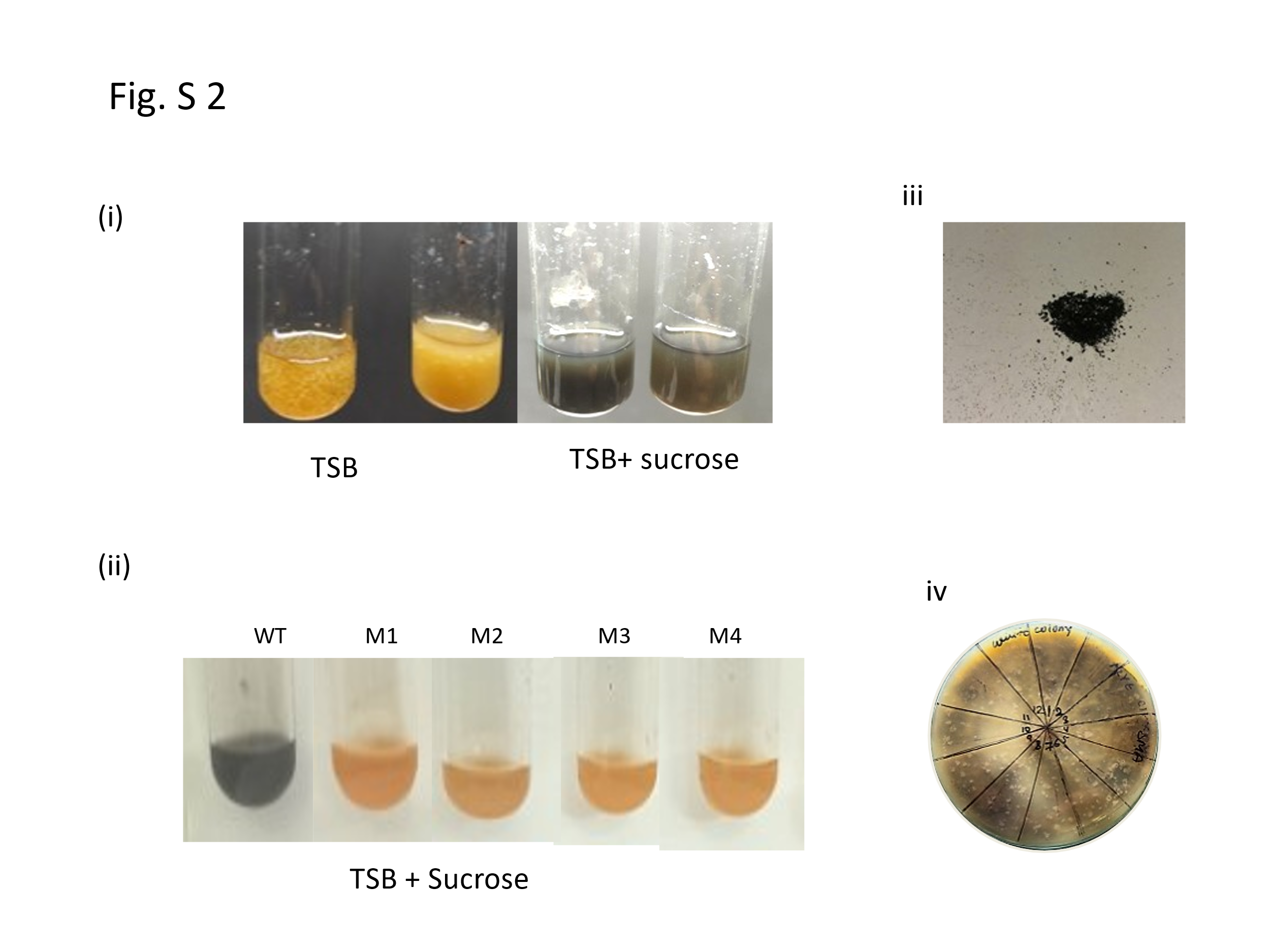


i) The diffusible black/brown pigment produced in the cells cultured in high osmolarity (TSB+Sucrose) medium (ii) can be precipitated by 6N HCl and purified by washing with water. (iii) Black pigment non-producer mutant isolated by UV mutagenesis (M1 through M4) don’t produce diffusible pigment in TSB+sucrose medium and are not affected for viability (not shown). (iv) Both wild-type and mutants produce additional brown/lack diffusible pigment on Soyabean Mannitol Agar (SMA).

**Figure S3: Reason for selecting *pfk2* as an internal control/reference gene in quantitative real time PCR.**


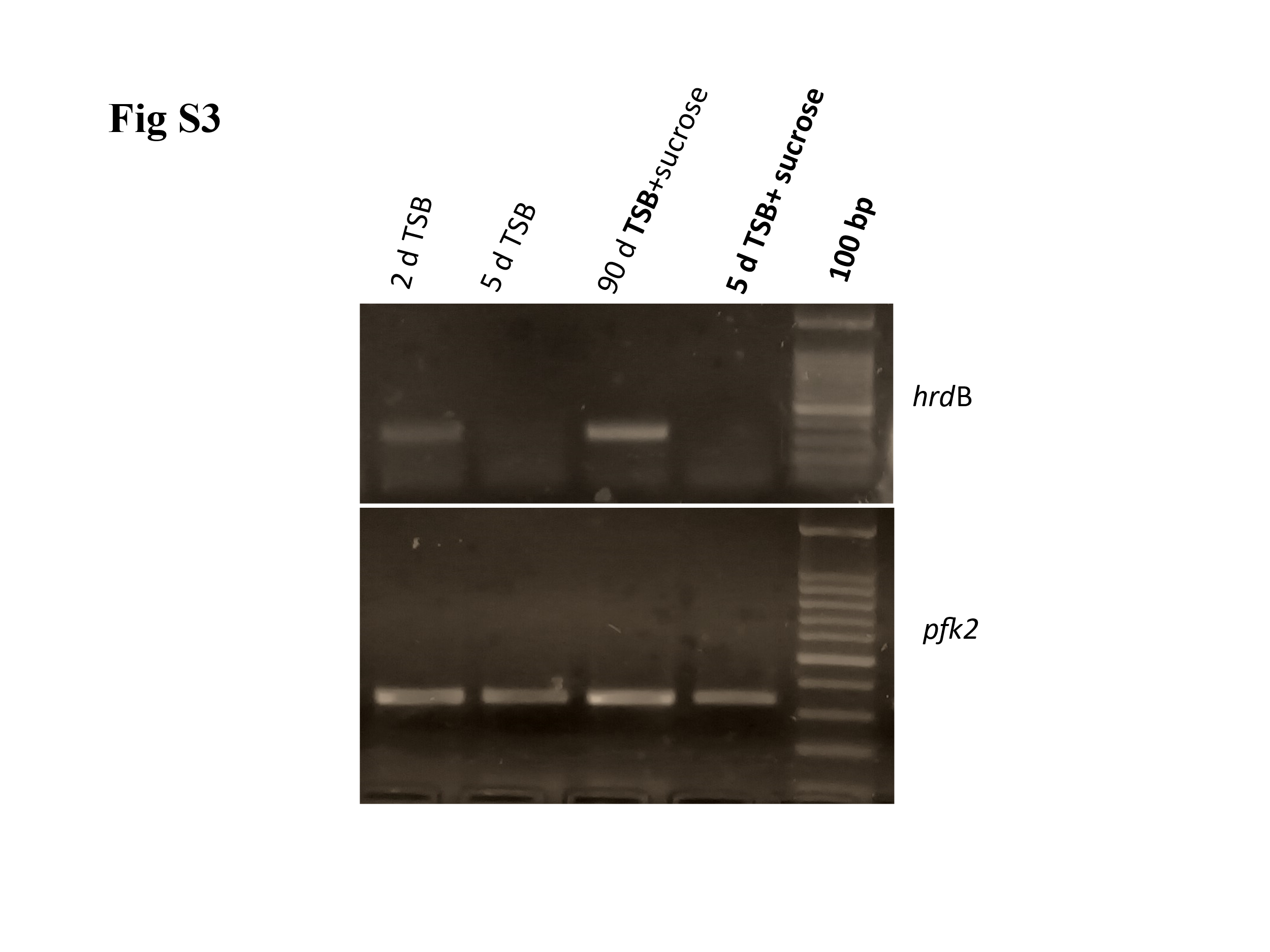


Amounts of *hrdB* transcript exhibited variation in the cells grown for different days in media of varying osmolarity compared to the consistent level of *pfk2* transcript. A representative image of the qualitative analysis of the transcript is shown.

**Figure S4: Loss of viability of *clp*X mutant (JP4) cells grown in high osmolar medium (TSB+sucrose between 20-24 days in the stationary phase**


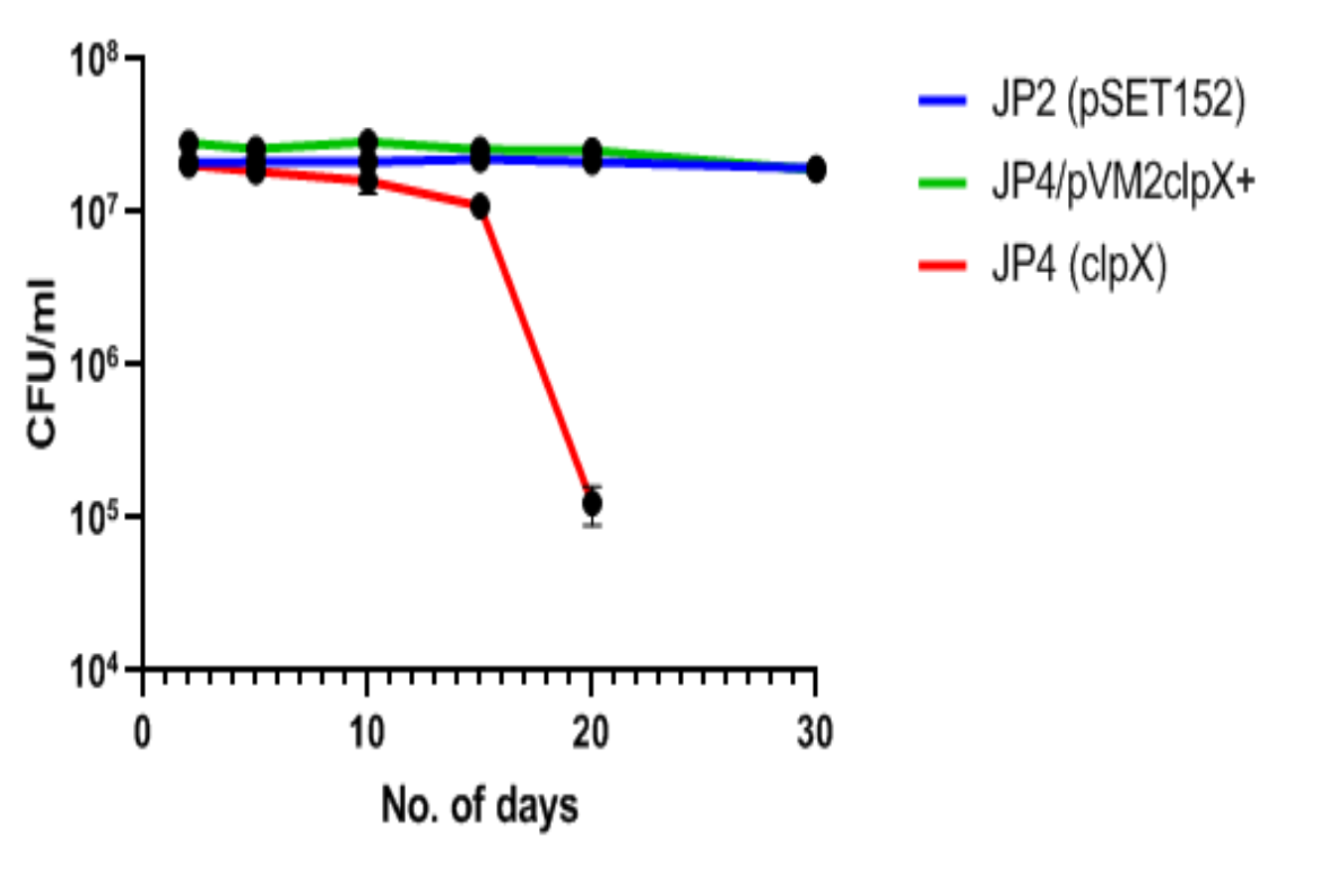


Viability as measured by CFU count decreases in the cells of *clpX* mutant (JP4) cultivated in high osmolarity TSB+sucrose medium between 20-24 days. This experiment is representative of two independent trials.

**Figure S5: Loss of viability of *relA* mutant cells (JP3) cultivated in high osmolar (TSB+sucrose) medium between 15-18 days in the stationary phase**


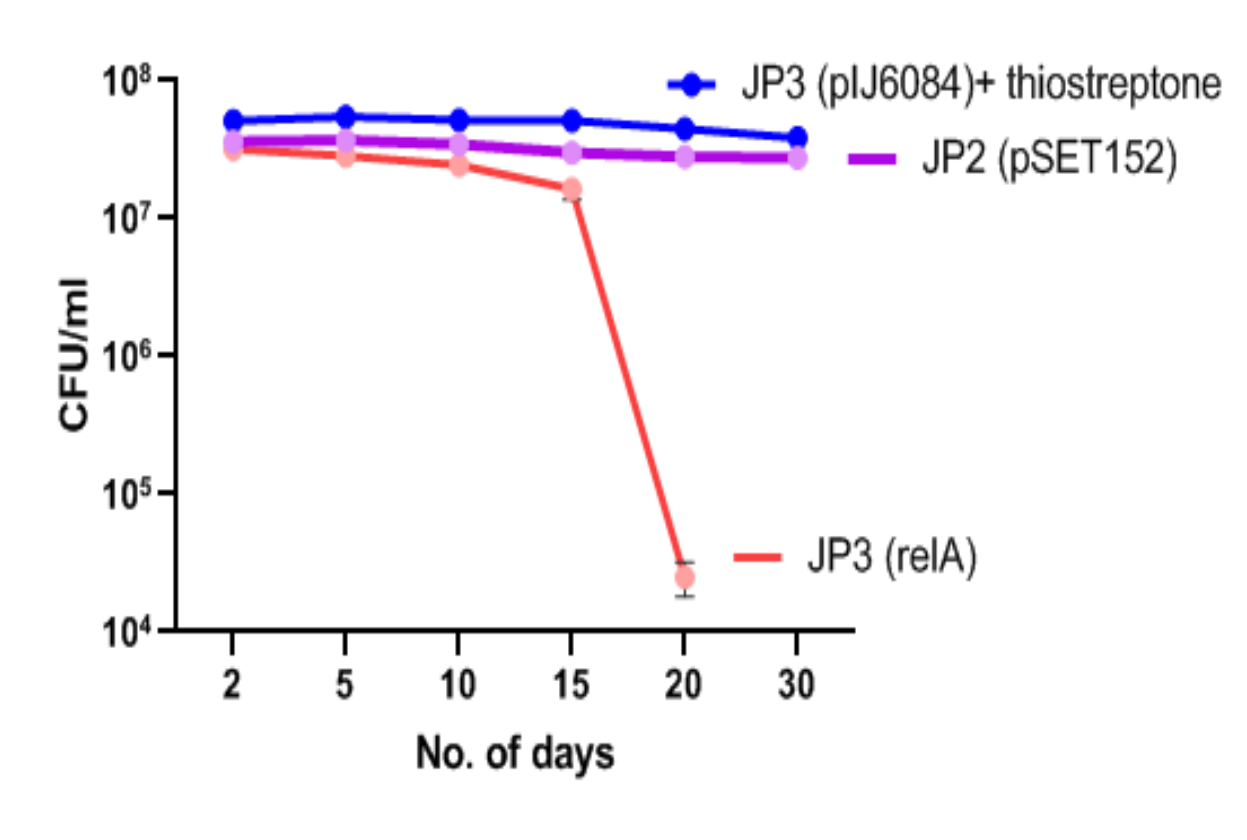


Viability, as measured by CFU count, decreases in the cells of *relA* mutant (JP3) cultured in high osmolarity medium (TSB+sucrose) between 15-18 days in comparison to the cells of JP2 and JP3(pIJ6084). The latter strain was supplemented with 10 µg/ml of thiostrepton during growth. This experiment is representative of two independent experiments.

**Table S1: Strains and plasmids used in this study**

| S.No. | *Escherichia.coli* | Genotype | Reference |
| --- | --- | --- | --- |
| 1 | DH5α | *fhuA2 lac(del)U169 phoA glnV44 Φ80' lacZ(del)M15 gyrA96 recA1 relA1 endA1 thi-1 hsdR17* | Lab collection |
| 2 | ET12567/pUZ8002 | F- *dam-13*::Tn*9* *dcm-6 hsdM hsdR zjj-202*::Tn*10* *recF143 galK2 galT22 ara-14 lacY1* *xyl-5 leuB6 thi-1 tonA31* *rpsL136 hisG4* *tsx-78 mtl-1 glnV44* | John Innes Centre  (JIC) , U.K. |
| ***Streptomyces* strains** | | | |
| 1. | *S.minutiscleroticus* (WT) | PKS^+^ Produces yellow coloured antibiotic chromomycin | MTCC, IMTECH,  Chandigarh, India |
| 2. | JP2 (WT Control) | *attC*::pSET152 | This work |
| 3 | JP3 | *relA*::pVM1’*relA*’ | This work |
| 4 | JP4 | *clpX*::pVM1’*clpX*’ | This work |
| 5. | JP5 | WT/pIJ12551 | This work |
| 6. | JP6 | WT/pIJ8655 | This work |
| **Plasmids** | | | |
| 1. | pSET152 | φC31*int* gene*, aac(3)IV* gene*, lacZα* gene*, oriT, colE1* origin, size- 5.7kb, *Streptomyces* integrating vector | John Innes Centre  (JIC), U.K. |
| 2. | pVM1 | Removal of 1.8 kb *int* DNA by *Hind*III restriction enzyme digestion of pSET152 followed by re-circularization, size-3.9 kb. | This work |
| 3 | pIJ12551 | *aac(3)IV*, size- 6064 bp, φC31 *Int* gene, *oriT*, conjugative, integrative plasmid, *ermE**p | John Innes Centre  (JIC), U.K. |
| 4 | pIJ10257 | φBT1*int* gene, size- 6.6 kb, conjugative, integrative plasmid, *oriT*, Hygromycin resistance gene, *ermE**p | John Innes Centre  (JIC), U.K. |
| 5 | pVM2 | *clpX* full length PCR of 1.4 kb cloned in restriction sites *Hind*III and *Nde*I of pIJ10257. | This work |
| 6 | pVM3 | 1.5 kb *sasA*8 full length PCR cloned in pIJ12551 at *Nde*I and *Pac*I restriction enzyme sites. | This work |
| 7 | pIJ8655 | *aac(3)IV*, φC31 *Int*, *ori*T, eGFP gene under (thiostreptone inducible promoter) tipA_p_ | John Innes Centre  (JIC), U.K. |
| 8 | pIJ8600 | *aac(3)IV*, size – 8.1 kb, φC31 *Int*, *ori*T, tipA_p,_ tsr | John Innes Centre  (JIC), U.K. |
| 9 | pIJ6084 | Thiostrepton-inducible expression vector, 1.46 kb C terminal truncated *Not*I- *Pvu*II *rel*A fragment of pIJ8625 cloned with suitable modifications in pIJ8600, tsr, *aac(3)IV*, φC31 *Int*, *ori*T. | John Innes Centre  (JIC), U.K. |

**Table S2: List of primers used in the study**

| S.No. | Primers Name | Sequence |
| --- | --- | --- |
| 1 | Sas A Left  Sas A Right | GAGGGTGGTGTTGATGGTG  CATGGTCATGTCCACCGATA |
| 2 | rplJ Left  rplJ Right | TCCGCAAGCTTCTTGATCT  AGTTCCGCAACTCGAACG |
| 3 | rpsG Left  rpsG Right | CGAGAAGCTCGTTGAGCAG  TACGGCTCTCCTCTGGTGAC |
| 4 | atpB Left  atpB Right | ACGATCGACCAGAGGTTCAT  AGACCGACTGCCACATCTTC |
| 5 | arc Left  arcRight | TCTTCGGCAAGTACCTCACC  CCTCGTTCTCCTTGAACTCG |
| 6 | Rsbw Left  Rsbw Right | GCTGTTTGTAGAGGCTGATCG  GACCCAGGACTTCGTGGAA |
| 7 | tuf_1 Left  tuf_1 Right | GTTGTTGAAGAACGGGGTGT  AGGACGTCTTCACGATCACC |
| 8 | clpP2 Left  clpP2 Right | ACACGATGCAGTTCGTGAAG  GACCAGACCGTACGACAGTG |
| 9 | hpf1 left  hpf1 right | AATGGATCCGATCTTCTTGACCGGTACGC  AATGAATTCGCTGGAGAAGATCCAGAAGC |
| 10 | relA left  relA right | AATGGATCCGCGATGTAGTCCTTGAACC  AATGAATTCGTGACCAAGCTGGACAAGGT |
| 11 | clp2 left  clp2 right | AATGGATCCGACATCGCGAACAAGATCAC  AATGAATTCGCTTCTTGGTGTGCAGCAG |
| 12 | hppd left  hppd right | \| AATGGATCCGAAGCCCATGACCTTGTTGT \| \| --- \| \| AATGAATTCGTGTTCACCTCCGTCATCAA \| |
| 13 | hypo 1 left  hypo 1 right | TTCAGGAAGGTGACCTCGAC  CACGGTACGGGAGCTGAT |
| 14 | HTH left  HTH right | CCATGACGCAAGAAGATGG  CTGACGCATCACGCTCATAC |
| 15 | trap left  trap right | GTACAGCCACAGCTCCTTGG  TCCCCGACTACACCTACGTC |
| 16 | clp first left  clp first right | AATCATATGCGTAAGACGAGCAGGTGGAT  GACCAGACCGTACGACAGTG |
| 17 | clp3 gd left  clp3 gd right | AATGAATTCAGCCAGAAGCAGGTCAAGAA  AATGAATTCGCCTCGATGATCTTCTCCAG |
| 18 | sod left  sod right | AATGGATCCCTGGTGGTCGTAGACCTGCT  AATGAATTCAGATCATCGAGCTGCACCA |
| 19 | SDH LEFT  SDH RIGHT | AATGGATCCAAGTACTGGCCGTCGTTCC  AATGAATTCAAGATCAAGTGGGACCTGGA |
| 20 | clp second left  clp second right | CACTGTCGTACGGTCTGGTC  AATGGATCCGGTTACGCCGACTTCTGCT |
| 21 | DUF4132 left  DUF4132 right | GGGACGATGCAGAGGTATTC  CTCGTCCTCTCCGAAGTCC |
| 22 | sasA left cloning  sasA right cloning | AATGGATCCTTAATTAAGTCGAGCGCGTCATTTCT  AATCATATGACCGTACGAGGTATCGGCTA |
| 23 | \| hth left cloning \| \| --- \| \| hth right cloning \| | \| AATTTAATTAAGTCATGGCATCACCGTAAAC \| \| --- \| \| AATCATATGCTCTCGTAGGGGGAGGTCTG \| |
| 24 | \| arc left cloning \| \| --- \| \| arc rgt cloning \| | \| AAT GGATCCACGAGGATTCTCGAAGAGC \| \| --- \| \| AATGAATTCGGGACGACCTCGTAGACGTA \| |
| 25 | tyr int left  tyr int right | CACAAGAAGTGGGTGGTCAA  GAGTGGTGCAGCCAGAAGAC |
| 26 | lysr left  lysr right | AATGGATCCGAGTCCGGCAGCTTCACC  AATGAATTCGGGTGGTGACCGTGATCT |
| 27 | duf cloning left  duf cloning right | AATGGATCCGACCCGTACAACGCCAAC  AATGAATTCTTGAGCTCCTTCAGCTCCTC |
| 28 | alcohol left  alcohol right | AATGGATCCGCTGACGAGCAGTGTCTCAC  AATGAATTCGTGAAGTGCTGGTCGAGGTG |
| 29 | alanine left  alanine right | AATGGATCCGCACGCCTTCTCCAGTTC  AATGAATTCACCTGCTGCTGAAGGTCAAG |
| 30 | gapdh left  gapdh right | AATGGATCCGGTCGTACTGGTCCTGGTTG  AATGAATTCACTTCGGAGCTGGAGATCGT |
| 31 | Cytbd left  Cytbd right | AATGGATCCGGGTCCTCATCAACACCATC  AATGAATTCAACAGCGTCAGTGTCACCAG |
| 32 | fumarate left  fumarate right | AATGGATCCGTCTGGCGGCAGAAGAAC  AATGAATTCCTGGATGATGCGGTCGAAG |
| 33 | clpX rt Left  clpX rt right | GAGTTGGCGAAGTCCAACAT  GTCGTGTCGATCTGGATGAA |

**Table S2: List of primers used in the study (continued)**

**Table S3: List of selected genes whose expression level was upregulated by log2 fold 2 and above (submitted separately).**

**Table S4: Specific activities of the enzymes by biochemical methods**

| S.No | Protein function | Log2 fold change | Enzyme activity (U/  mg protein) in 5 days low osmolarity grown cells | Enzyme activity (U/mg protein) in 90 days high osmolarity grown cells | Enzyme activity (U/mg protein) in 2 days low osmolarity grown cells |
| --- | --- | --- | --- | --- | --- |
| 1 | L-alanine dehydrogenase | 4.27 | 0.7  ±0.09 | 1.5  ±0.02 | 1.3  ±0.12 |
| 2 | Citrate synthase | 4.77 | 0.84  ±0.02 | 2.47  ± 0.05 | 2.81  ±0.18 |
| 3 | Fumerate reductase | 3.46 | 0.67  ±0.12 | 1.28  ±0.09 | 1.69  ±0.05 |
| 4 | GAPDH activity | 5.76 | 1.10  ±0.03 | 3.35  ±0.26 | 3.30  ±0.13 |
| 5 | Succinate co-A ligase | 5.12 | 1.02  ±0.14 | 3.74  ±0.21 | 2.98  ±0.09 |
| 6 | Succinate dehydrogenase | 2.62 | 0.70  ± 0.06 | 2.96  ±0.66 | 2.21  ±0.20 |
| 7 | SOD assay | 5.88 | 4.85  ±0.02 | 6.54  ±0.07 | 5.95  ±0.21 |
| 8 | Catalase assay | 3.04 | 12.5  SD±0.91 | 21.8  SD±0.008 | 13.5  SD± 0.42 |

**Table S5: List of functions targeted for mutagenesis to study their effect on viability of LTSP cells.**

| **Accession No** | **Log2 fold** | **Knock out Mutation in** | **Putative function** | | **Viability phenotype^a^** |
| --- | --- | --- | --- | --- | --- |
| A0A918U6D2 | 7.94 | *arc* | Proteasome-associated ATPase | | Viable |
| A0A918U6R3 | 4.23 | *ald_*1 | Alanine dehydrogenase | | Viable |
| A0A918K833 | 5.88 | *sodF1_*1 | Superoxide dismutase [Fe-Zn] 1 | | Viable |
| A0A918NZ26 | 5.76 | *gap2_*1 | Glyceraldehyde-3-phosphate dehydrogenase 2 | | Viable |
| A0A918KML7 | 14.17 | Hypothetical protein | DUF4132 domain containing protein | | Viable |
| A0A918NI07 | 12.39 | Hypothetical protein | Helix-turn-helix protein in S.minutscleroticus | | Viable |
| A0A918KN69 | 2.62 | \| *sdh*B \| \| --- \| | fumarate reductase iron-sulfur subunit | | Viable |
| A0A918KQF9 | 13.72 | *sasA_*8 | Adaptive-response sensory-kinase SasA | | Viable |
| \| A0A918K6D7 \| \| --- \| \|  \| | 2.90 | *clpX_*2 | ATP-dependent Clp protease ATP-binding subunit ClpX | | Viability loss after 25 days |
| A0A918NQJ4\| | 3.46 | *frd*B | Succinate dehydrogenase iron-sulfur subunit | | Viable |
| A0A918K7D6 | 4.65 | *clpP1_*2 | ATP-dependent Clp protease proteolytic subunit 1 | | Viable |
| A0A918NMY0 | 1.94 | *cyd*B | Cytochrome c oxidase assembly protein | | Viable |
| A0A918NHZ6 | 12.15 | Hypothetical protein | hypothetical protein | | Viable |
| A0A918NY76 | 6.59 | *hpf* _1 | Hibernation promoting factor | | Viable |
| **Mutation in other genes-** | | | | | |
| A0A918N579 |  | *rel*A*/spo*T | (p)ppGpp synthatase/GTP pyrophosphokinase | Viability loss after 20 days | |
| A0A918KKK9 |  | *hpd* | 4-hydroxyphenylpyruvate dioxygenase**^b^** | Viable, no effect on pigment production | |
| A0A918NL47 |  |  | 1,3,6,8-tetrahydroxynaphthalene synthase**^b^** | Viable, no effect on pigment production | |
| A0A918KK45 |  | *tyr* | Tyrosinase (monophenol monooxygenase)**^b^** | Viable, no effect on pigment production | |

**^a^** – Number of CFU is same as that of non-mutant WT on day 30

**^b^** - Mutation in the putative genes to test effect on synthesis of black/brown diffusible pigment.

**Biochemical enzymes assays -**

**Alanine dehydrogenase** – 1 ml cells were harvested from cultures grown for 2days (TSB), 5days (TSB) and 90 days (TSB + Sucrose) medium and washed twice with N-saline. These cells were sonicated for 1minute (20 sec pulse on, 5 sec pulse off at 20% amplitude) and the cells were spin down at 10000 rpm for 10 min at 4°C. The cell lysate was used for the assay. The reaction mixture contained 50 mM Sodium Carbonate Buffer, pH 10.0, 500 mM L-Alanine Solution, 30 mM ß-Nicotinamide Adenine Dinucleotide and the cell extract. Enzyme activity recorded by the increase in *A*_340nm_ for approximately 5 minutes and calculated by the formula (4).

L-Alanine + ß-NAD + H2O L-Alanine Dehydrogenase> Pyruvate + ß-NADH + NH3

Units/ml enzyme = (ΔA 340nm /min Test - ΔA340nm /min Blank)(1)(df)/ (6.22)(0.1)

Units/mg protein = (units/ml enzyme)/(mg protein/ml enzyme)

1 = Volume (in milliliters) of assay

df = Dilution factor

6.22 = Millimolar extinction coefficient of ß-NADH at 340 nm

0.1 = Volume (in milliliter) of enzyme used

One unit convert 1.0 μmole of L-alanine to pyruvate and NH3 per minute at pH 10.0 at 25°C.

**Citrate synthase activity**- 1 ml cells were washed with N-saline and sonicated for 1min. The cell extract (enzyme solution) was incubated at 25°C with 0.2mM oxaloacetate, 0.15mM acetyl Co-A in 20mM Tris/HCl (PH 8)/1mM- EDTA. The samples were withdrawn after every 1min for 5 min and added to 0.45ml of 0.5 mM DNPH solution prepared in 1M- HCl. This solution was left for 10 min and then 0.5ml of 3M NaOH was added to the solution. After 5 min A_450_ was recorded. Specific activity was expressed as units /mg protein (2).

**Fumarate reductase activity** - Fumarate reductase activity was measured by addition of cell extract in reaction mixture contained 50 mM phosphate buffer (pH 6.5), 20 mM fumarate, and 0.2 mM NADH at 30°C. Decrease in absorbance and oxidation of NADH was measured at 340nm. The enzyme activity was denoted as the oxidation of 1 μmol NADH per milligram of total protein per minute (3).

**Succinate dehydrogenase activity**- Succinate dehydrogenase activity was measured at 630 nm. The reaction contains 100mM phosphate buffer (pH 7.4), 0.1 M NBT solution in 5% methanol, sodium succinate (100mM) and cell lysate (enzyme), for stop reaction 2% SDS was added. Enzyme activity was defined by units/mg protein.

**GAPDH assay**- GAPDH activity was measured spectrophotometrically at 340 nm. The assay mixture contains 50mM Tris chloride buffer (pH-8.0), 4 mM mercaptoethanol, 4mM EDTA, 2mM NAD^+^ reaction started by the addition of glyceraldehyde 3 phosphate as a substrate at the final concentration of 0.4mM. Specific enzyme activity is measured as units /mg protein.

**Succinyl Co-A ligase activity-** The reaction mixture for succinyl Co-A ligase activity contained 50 mM Tris/Hcl buffer (pH 7.2), 100 mM KCl, 10 mM MgCl_2_, 0.4mM ATP, 0.1 mM Co enzyme A, 20 mM sodium succinate and the cell lysate as enzyme. Absorbance is taken at 230 nm. Specific enzyme activity was expressed as units per/mg protein.

**SOD-**Superoxide dismutase activity was measured by pyrogallol method. Different volumes of cell supernatant were added to 0.1 mM TrisCl buffer of pH 8.0 followed by 100 μl of 4 mM of pyrogallol. Autooxidation of pyrogallol was monitored by recording the absorbance at 420 nm every 10 sec for 3 minutes. Specific activity of SOD was estimated by units of enzyme required for 50% inhibition of auto-oxidation of pyrogallol per mg protein.

**Catalase activity** -was quantified spectrophotometrically by following the rate of decrease

in absorbance at 240 nm caused by the disappearance of H2O2. The assay mix contained protein extract and 10 mM of H2O2 in 50 mM phosphate buffer pH 7.4 to final volume of 1

ml. Assay carried out at 25^°^ C. One unit of enzyme activity is defined as the amount required for the conversion of 1μmol substrate into product per minute.

**References:**

1. T. Kieser, D. A. Hopwood, K. F Chater., M. J. Buttner, & M. J Bibb, Practical Streptomyces.  Genetics. Norwich, UK: John Innes Centre (2000).
2. R. Muthuraj , M.S. Kumar , R.S. Kumar ,  A.I. Abdulrahman, P. Karthikeyan, N.Sreenivasa,  Bioproduction, purification and physicochemical characterization of melanin from Streptomyces sp. strain MR28. [Microbiol. Res](https://www.sciencedirect.com/journal/microbiological-research)[. 263](https://www.sciencedirect.com/journal/microbiological-research/vol/263/suppl/C), 127130 (2022).
3. M. E. Pavan, N. I. López & M. J. Pettinari, Melanin biosynthesis in bacteria, regulation and production perspectives. Appl. Microbiol. Biotechnol. 104, 1357-1370 (2019).
4. KE. Keith, L. Killip, P. He, GR. Moran, MA. Valvano, Burkholderiacenopacia C5424 produces a pigment with antioxidant properties using a homogentisate intermediate. J. Bacteriol. 189, 9057-9065 (2007).
5. J. Else, S. J. Barnes, M. J. Dansont, P.D.J. Weitzman, A new spectrophotometric assay for citrate synthase and its use to assess the inhibitory effects of palmitoyl thioesters. Biochem. J. 251, 803-807 (1988)
6. L. Yang,  M. Lübeck,  B. K. Ahring , P. S. Lübeck, Enhanced succinic acid production in  Aspergillus saccharolyticus by heterologous expression of fumarate reductase from Trypanosoma brucei. Appl. Microbiol. Biotechnol. 100, 1799–1809 (2016)
7. H.U. Bergmeyer, Methods of Enzymatic Analysis, 2nd edition. Volume I, 427 (1983).
